# Anti-Inflammatory, Neurotrophic, and Cytotoxic Oxylipins Isolated from *Chaenomeles sinensis* Twigs

**DOI:** 10.3390/antiox12020284

**Published:** 2023-01-27

**Authors:** Da Yeong Lee, Kyoung Jin Park, Lalita Subedi, Gyu Sung Lee, Ji-Hyeok Lee, Won-Min Lee, Sang Un Choi, Seong-Min Hong, Sun Yeou Kim, Chung Sub Kim

**Affiliations:** 1 Department of Biopharmaceutical Convergence, Sungkyunkwan University, Suwon 16419, Republic of Korea; 2 School of Pharmacy, Sungkyunkwan University, Suwon 16419, Republic of Korea; 3 Gachon Institute of Pharmaceutical Science, Gachon University, Incheon 21936, Republic of Korea; 4 Department of Biohealth Regulatory Science, Sungkyunkwan University, Suwon 16419, Republic of Korea; 5 Korea Research Institute of Chemical Technology, Daejeon 34114, Republic of Korea; 6 College of Pharmacy, Gachon University, Yeonsu-gu, Incheon 21936, Republic of Korea

**Keywords:** *Chaenomeles sinensis*, oxylipins, anti-inflammation, neurotrophic effect, cytotoxicity

## Abstract

Oxylipins are important biological molecules with diverse roles in human and plants such as pro-/anti-inflammatory, antimicrobial, and regulatory activity. Although there is an increasing number of plant-derived oxylipins, most of their physiological roles in humans remain unclear. Here, we describe the isolation, identification, and biological activities of four new oxylipins, chaenomesters A–D (**1–4**), along with a known compound (**5**), obtained from *Chaenomeles sinensis* twigs. Their chemical structures were determined by spectroscopic (i.e., NMR) and spectrometric (i.e., HRMS) data analysis including ^1^H NMR-based empirical rules and homonuclear-decoupled ^1^H NMR experiments. Chaenomester D (**4**), an omega-3 oxylipin, showed a potent inhibitory effect on nitric oxide (NO) production in lipopolysaccharide (LPS)-activated BV-2 cells (NO production, 8.46 ± 0.68 μM), neurotrophic activity in C6 cells through the induction of the secretion of nerve growth factor (NGF, 157.7 ± 2.4%), and cytotoxicity in A549 human cancer cell lines (IC_50_ = 27.4 μM).

## 1. Introduction

Oxylipins are a family of oxygenated fatty acids found in diverse living organisms such as fungi, plants, and animals. In general, oxylipins are classified into two categories, omega-3 and omega-6. Interestingly, while the only difference in the chemical structure of omega-3 and 6 oxylipins is the existence of an additional double bond in omega-3 compared to omega-6 molecules, they have opposite actions in the inflammatory response [1]. Omega-3 oxylipins, such as eicosapentaenoic acid (EPA) and docosahexaenoic acid (DHA), are anti-inflammatory, whereas omega-6 oxylipins are pro-inflammatory [2]. Non-steroidal anti-inflammatory drugs (NSAIDs) are one of the most well-known and widely used medicines which inhibit the biosynthesis of inflammation-related prostaglandins from omega-6 oxylipin arachidonic acid by blocking human cyclooxygenases (COX) [3,4,5,6]. In plants, jasmonic acid, synthesized from omega-3 α-linolenic acid, is a plant hormone regulating plant responses to abiotic and biotic stress as well as plant growth and development [7].

**Figure 1:**
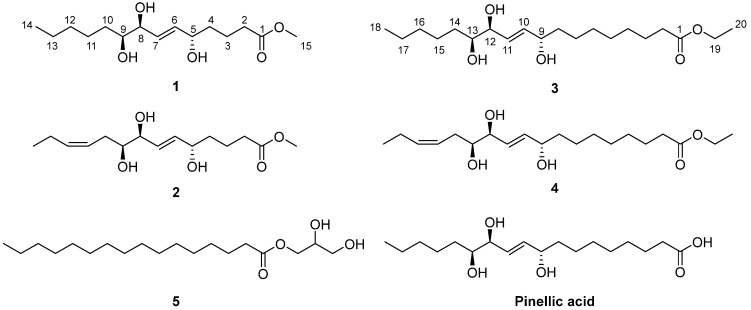
Chemical structures of the isolated compounds **1–5** and pinellic acid.

Given the pharmaceutical and biological importance of the naturally occurring oxylipins, our groups previously reported short- and long-chain oxylipins with anti-inflammatory, neurotrophic, and/or cytotoxic activities from several Korean traditional medicinal plants, *Chaenomeles sinensis* Koehne [8], *Wasabia japonica* (Miq.) Matsum. [9], *Sorbus commixta* Hedl. [10], and *Hosta longipes* (Fr. et Sav.) Matsum. [11]. *C. sinensis* (Rosaceae) is a semi-evergreen tree widely distributed in Korea, Japan, and mainland China. Its fruit has been used as a Korean traditional medicine for over 400 years for the treatment of myalgia, beriberi, diarrhea, and vomiting [12]. In our previous study on *C. sinensis* twigs, we isolated 11 oxylipins from the ethyl acetate (EtOAc)-soluble fraction of a *C. sinensis* MeOH extract, and they showed moderate anti-inflammatory activity by inhibiting nitric oxide (NO) production from lipopolysaccharide (LPS)-induced BV-2 microglia cells [8]. To find more potent anti-inflammatory oxylipins from *C. sinensis*, we further investigated its chloroform (CHCl_3_) and *n*-butanol (*n*-BuOH) fractions. Herein, we report the isolation, structure characterization, and biological activities of four new oxylipins, chaenomesters A–D (**1–4**), along with a known compound (**5**) from *C. sinensis* twigs (Figure 1). Their chemical structures were elucidated by an intensive analysis of NMR and HRMS data. Among the isolated compounds, the new compound chaenomester D (**4**) showed potent anti-inflammatory, neurotrophic, and cytotoxic activities.

## 2. Materials and Methods

### 2.1. General Experimental Procedures

Specific rotations were measured using a JASCO P-1020 polarimeter (JASCO, Easton, MD, USA) at 590 nm. We acquired 1D (^1^H and ^13^C) and 2D (COSY, HSQC, and HMBC) NMR spectra on a Bruker AVANCE III 700 NMR spectrometer (Bruker, Karlsruhe, Germany) at 700 MHz (^1^H) and 175 MHz (^13^C); chemical shifts are given in ppm (*δ*). Deuterated solvents (chloroform-*d* and methanol-*d*_4_) (Cambridge Isotope Laboratory, Inc., Tewksbury, MA, USA) were used for the NMR measurements of the purified compounds. The resultant spectra were analyzed using MestReNova (Mnova, version 14.1.2-25024). High-resolution fast atom bombardment mass spectroscopy (HRFABMS) spectra were obtained on either a JEOL JMS700 (Tokyo, Japan) or a Waters SYNAPT G2 (Milford, MA, USA) mass spectrometer. The separation of the fractions was performed through column chromatography, employing either a silica gel 60 (70−230 and 230−400 mesh; Merck, Darmstadt, Germany) or an RP-C_18_ silica gel (230−400 mesh; Merck, Darmstadt, Germany). The purification of the compounds was carried out on a high-performance liquid chromatographer (HPLC) furnished with a Shodex refractive index detector (Gilson, New York, NY, USA) and a Gilson 306 pump (Gilson, Middleton, WI, USA) using semipreparative conditions (flow rate: 2 mL/min; column: Phenomenex, Luna 10 μm C_18_ (2) 250 × 10 mm i.d.). Thin-layer chromatography (TLC) was performed on precoated silica gel F254 plates or RP-C_18_ F254s plates (Merck, Darmstadt, Germany). The spots on the TLC plates were visualized by irradiating UV light or heating after spraying with anisaldehyde−sulfuric acid.

### 2.2. Plant Material

Twigs of two-year-old *C. sinensis* (7.0 kg) were purchased in January 2012 from Yangjae Flower Market in Seoul, Korea. A voucher specimen of the plants (SKKU-NPL 1206) was authenticated by Prof. Kang Ro Lee and stored in the herbarium of the School of Pharmacy, Sungkyunkwan University, Suwon, Korea.

### 2.3. Compounds Extraction and Isolation

Twigs of *C. sinensis* (7.0 kg) were air-dried and chopped. The sample was extracted under reflux with 80% aqueous MeOH (10 L × 1 day, 3 times each) and the crude extract was filtered. The solvent in the filtrate was evaporated in vacuo to yield a total of 320 g of MeOH extract. The 80% MeOH extract was suspended in distilled water and successively partitioned with *n*-hexane, CHCl_3_, EtOAc, and *n*-BuOH to yield 3, 15, 6, and 30 g of fractions, respectively. Chromatographic separation of the CHCl_3_-soluble fraction (15 g) on a silica gel column (CHCl_3_–MeOH, 50:1 → 1:1) was first performed to afford 12 fractions (MGC1–MGC12). Fractions MGC5 (1.1 g) and MGC6 (0.4 g) were passaged over an RP-C_18_ silica gel column, eluted with 70% aqueous MeOH, to yield 12 subfractions (MGC5-1–MGC5-12). Compounds **3** (4 mg) and **4** (5 mg) were acquired by purifying the sub-fractions MGC5-5 (40 mg) and MGC5-6 (19 mg), respectively, employing a semipreparative RP-C_18_ silica HPLC system eluting with an isocratic 75% aqueous MeOH. Fractions MGC3 and MGC4 were combined (total 1.6 g) and then fractionated by an RP-C_18_ silica gel column (60% aqueous MeOH) and further purified by a semipreparative RP-C_18_ silica HPLC system (80% aqueous MeCN) to obtain compound **5** (16 mg). Subsequently, the *n*-BuOH-soluble fraction (30 g) was separated using silica gel column chromatography, and 10 subfractions (MGB1–MGB10) were obtained by eluting with CHCl_3_–MeOH–H_2_O (3:1:0.1). From the fraction MGB1 (30 mg), compounds **1** (2 mg) and **2** (2 mg) were acquired by utilizing a C_18_ Waters Sep-Pak cartridge with 60% MeOH and following semipreparative HPLC purification with aqueous 60% MeOH as an eluent.

*Chaenomester A* (**1**). Colorless gum; ${\left[\alpha \right]}_{D}^{25}$ +20 (c 0.1, MeOH); ^1^H (700 MHz) and ^13^C NMR (175 MHz) data in methanol-*d*_4_, see Table 1; HRFABMS (positive-ion mode) *m/z* 311.1829 [M + Na]^+^ (calcd. for C_15_H_28_O_5_Na^+^, 311.1829).

*Chaenomester B* (**2**). Colorless gum; ${\left[\alpha \right]}_{D}^{25}$ −130 (c 0.1, MeOH); ^1^H (700 MHz) and ^13^C NMR (175 MHz) data in methanol-*d*_4_, see Table 1; HRFABMS (positive-ion mode) *m/z* 309.1672 [M + Na]^+^ (calcd. for C_15_H_26_O_5_Na^+^, 309.1672).

*Chaenomester C* (**3**). Colorless gum; ${\left[\alpha \right]}_{D}^{25}$ −12 (c 0.25, CHCl_3_); ^1^H (700 MHz) and ^13^C NMR (175 MHz) data in chloroform-*d*, see Table 1; HRFABMS (positive-ion mode) *m/z* 381.2612 [M + Na]^+^ (calcd. for C_20_H_38_O_5_Na^+^, 381.2611).

*Chaenomester D* (**4**). Colorless gum; ${\left[\alpha \right]}_{D}^{25}$ +10 (c 0.2, CHCl_3_); ^1^H (700 MHz) and ^13^C NMR (175 MHz) data in chloroform-*d*, see Table 1; HRFABMS (positive-ion mode) *m/z* 379.2455 [M + Na]^+^ (calcd. for C_20_H_36_O_5_Na^+^, 379.2455).

**Table 1:** ^1^H and ^13^C NMR spectroscopic data for compounds **1** and **2** in methanol-*d*_4_ and **3** and **4** in chloroform-*d*.

Pos.	1		2		3		4	
	*δ*_C_	*δ*_H_, multi., (*J* in Hz)	*δ*_C_	*δ*_H_, multi., (*J* in Hz)	*δ*_C_	*δ*_H_, multi., (*J* in Hz)	*δ*_C_	*δ*_H_, multi., (*J* in Hz)
1	175.8		175.8		174.1		174.1	
2	34.7	2.35, t (7.4)	34.7	2.35, t (7.4)	34.4	2.28, t (7.4)	34.4	2.28, t (7.4)
3	22.1	1.66, m; 1.71, m	22.2	1.66, m; 1.72, m	25.0	1.61, quin (7.4)	25.0	1.61, m
4	37.6	1.54, m	37.6	1.53, m	29.2	1.30 *	29.2	1.30 *
5	72.7	4.07, m	72.6	4.06, m	29.3	1.30 *	29.3	1.30 *
6	136.3	5.72, dd (15.5, 5.4)	136.2	5.72 *	29.1	1.30 *	29.1	1.30 *
7	131.4	5.69, dd (15.5, 5.5)	131.4	5.72 *	25.3	1.32 *; 1.39 *	25.3	1.32 *; 1.39 *
8	75.8	3.90, t (5.5)	75.7	3.95, m	37.3	1.53 *	37.3	1.54 *
9	76.5	3.41, m	75.9	3.45, m	72.2	4.15, q (5.9)	72.2	4.15, q (5.8)
10	33.6	1.35 *; 1.53 *	31.6	2.12, m; 2.35 *	136.4	5.82, dd (15.6, 5.9)	136.4	5.84, dd (15.5, 5.8)
11	26.7	1.34 *; 1.52 *	126.4	5.44, m	129.8	5.71, dd (15.6, 6.2)	129.7	5.73, dd (15.5, 6.0)
12	33.1	1.53 *	134.4	5.46, m	75.4	3.92, m	74.7	4.01, t (6.0)
13	23.8	1.33 *	21.7	2.06, quin (7.5)	74.7	3.47, m	74.2	3.53, m
14	14.5	0.91, t (7.1)	14.6	0.96, t (7.5)	33.1	1.42 *; 1.51 *	31.1	2.30, t (7.5)
15	52.0	3.65, s	52.0	3.65, s	25.4	1.38 *; 1.50 *	123.9	5.40, m
16					31.9	1.29 *	135.5	5.58, m
17					22.7	1.31 *	20.8	2.07, quin (7.5)
18					14.2	0.89, t (7.0)	14.3	0.97, t (7.5)
19					60.3	4.12, q (7.1)	60.3	4.12, q (7.1)
20					14.4	1.25, t (7.1)	14.4	1.25, t (7.1)

* Overlapped peaks.

### 2.4. Nitric Oxide (NO) Assay

BV-2 cells, developed by Dr. V. Bocchini, were employed to evaluate the anti-inflammatory effect of the isolated compounds [13]. The BV-2 microglioma cell line was used for measuring the produced levels of nitrite (NO_2_^-^) and maintained in Dulbecco’s modified Eagle’s medium (DMEM) containing 10% fetal bovine serum (FBS) and 1% penicillin–streptomycin (PS) in 5% CO_2_. The cells were seeded in a 96-well plate (4 × 10^4^ cells/well) and then treated with the tested compounds (**1**–**5**) at 20 µM for 30 min prior to treatment with 100 ng/mL of lipopolysaccharide (LPS) and incubation for 1 day. The produced NO_2_ levels, a soluble oxidized product of NO, was evaluated with Griess reagent including 0.1% *N*-1-napthylethylenediamine dihydrochloride and 1% sulfanilamide. The supernatant (50 µL) was mixed with an equal volume of Griess reagent. The absorbance was measured at 570 nm in 10 min. A graded sodium nitrite solution was used as a standard to determine the nitrite concentrations. In addition, the cytotoxicity of each compound was evaluated by the 3-[4,5-dimethylthiazol-2-yl]-2,5-diphenyltetrazolium bromide (MTT) assay at the same concentration, and we calculated the concentration of compound that caused a 50% inhibition of cell proliferation. All samples were dissolved in DMSO. Before treatment, the final concentration of 0.01% DMSO was used, which did not show cytotoxicity [14]. The reported nitric oxide synthase (NOS) inhibitor *N*^G^-monomethyl-L-arginine (L-NMMA) was used as a positive control.

### 2.5. Nerve Growth Factor (NGF) Assay

In order to measure the induction of nerve growth factor (NGF) release, C6 glioma cells were used and maintained in DMEM medium containing 10% fetal bovine serum (FBS) and 1% penicillin–streptomycin (PS) in 5% CO_2_. The tested cells were seeded onto a 24-well plate (1 × 10^5^ cells/well) and incubated for 1 day. The cells were treated with the isolated compounds at a 20 µM concentration (**1–5**) in DMSO, together with serum-free DMEM for 1 day. To measure the released NGF levels in the cell supernatants after treatment, an ELISA development kit (R&D System, Minneapolis, MN, USA) was used. In addition, an MTT assay was carried out to evaluate the cell viability in comparison with cells treated with 6-shogaol (positive control), and the results are presented as percentage viability with respect to the control group. The NGF secretion values for each treatment were divided by the respective cell viability values to calculate normalized NGF secretion levels which were further statistically analyzed with the Student’s *t*-test using GraphPad Prism 8. Statistical significance was considered for ^*^ *p* < 0.05, ^**^ *p* < 0.01, and ^***^ *p* < 0.001.

### 2.6. Cytotoxicity Assignment

The cytotoxic activity of the compounds (**1**–**5**) in four cultured human cancer cell lines was evaluated utilizing the sulforhodamine B colorimetric (SRB) method [15]. Each cell line was inoculated into standard 96-well flat-bottom microplates and incubated for 24 h at 37 °C in a humidified atmosphere with 5% CO_2_. The attached cells were incubated with the compounds serially diluted in DMSO (30, 10, 3, 1, 0.3, and 0.1 μM). After continuous exposure to these compounds for 48 h, the culture medium was removed, and the cells were fixed with 10% cold trichloroacetic acid at 4 °C for 1 h. After washing with tap water, the cells were stained with 0.4% SRB dye and incubated for 30 min at room temperature. These cells were washed again and then solubilized with a 10 mM unbuffered Tris base solution (pH 10.5). The absorbance was measured spectrophotometrically at 520 nm using a microtiter plate reader. The cell lines used for this study were A549 (non-small cell lung adenocarcinoma), SK-OV-3 (from an ovary malignant ascite), SK-MEL-2 (skin melanoma), and MKN-1 (adenosquamous carcinoma) and were purchased from the American Type Culture Collection (Manassas, VA, USA) and maintained at the Korea Research Institute of Chemical Technology. Etoposide (≥ 98%; Sigma Chemical Co., St. Louis, MO, USA) was used as a positive control.

## 3. Results and Discussion

### 3.1. Structure Elucidation of Compounds ***1**–**5***

**Figure 2:**
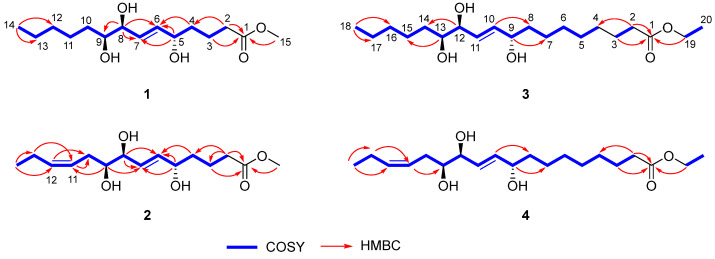
Key ^1^H-^1^H COSY and HMBC correlations of **1–4**.

Chaenomester A (**1**) was obtained as a colorless gum. The HRFAMMS analysis indicated that its molecular formula is C_15_H_28_O_5_ from the sodiated molecular ion peak [M + Na]^+^ at *m/z* 311.1829 (calcd. for C_15_H_28_O_5_Na^+^, *m/z* 311.1829). The ^1^H NMR data of **1** revealed the presence of a double bond [*δ*_H_ 5.72 (1H, dd, *J* = 15.5, 5.4 Hz, H-6) and 5.69 (1H, dd, *J* = 15.5, 5.5 Hz, H-7)], three oxygenated methines [*δ*_H_ 4.07 (1H, m, H-5), 3.90 (1H, t, *J* = 5.5 Hz, H-8), and 3.41 (1H, m, H-9)], a methoxy [*δ*_H_ 3.65 (3H, s)], seven methylenes [*δ*_H_ 2.35–1.33 (14H, H-2–H-4 and H-10–H-13)], and a methyl [*δ*_H_ 0.91 (3H, t, *J* = 7.1 Hz, H-14)] functionalities. The ^13^C NMR data displayed 15 peaks for a carbonyl [*δ*_C_ 175.8 (C-1)], two olefinic [*δ*_C_ 136.3 (C-6) and 131.4 (C-7)], three oxygenated [*δ*_C_ 76.5 (C-9), 75.8 (C-8), and 72.7 (C-5)], a methoxy [*δ*_C_ 52.0 (C-15)], and eight methylene/methyl [*δ*_C_ 37.6 (C-4), 34.7 (C-2), 33.6 (C-10), 33.1 (C-12), 26.7 (C-11), 23.8 (C-13), 22.1 (C-3), and 14.5 (C-14)] groups. These spectroscopic data were quite similar to those of pinellic acid methyl ester [16], except for the absence of four methylene signals in **1** compared to pinellic acid methyl ester, suggesting **1** to be a C14 oxylipin rather than a C18 oxylipin. We performed a 2D NMR analysis of **1**, including ^1^H-^1^H COSY, HSQC, and HMBC (Supplementary Materials), and the data were analyzed to elucidate the planar structure of **1**. The ^1^H-^1^H COSY correlations from H-2 to H-14 including two olefinic (H-6 and H-7) and three oxygenated methine protons (H-5, H-8, and H-9), and the HMBC cross-peaks of H-2/C-1 and C-4, H-3/C-1, H-4/C-6, H-5/C-6 and C-7, H-8/C-7, and C-9, and H-14/C-12 and C-13 confirmed a C14 oxylipin scaffold for **1** and the location of a double bond and three hydroxy groups (Figure 2). Additional HMBC correlation of H-15/C-1 indicated the methoxy group to be connected at C-1 through an ester bond.

**Figure 3:**
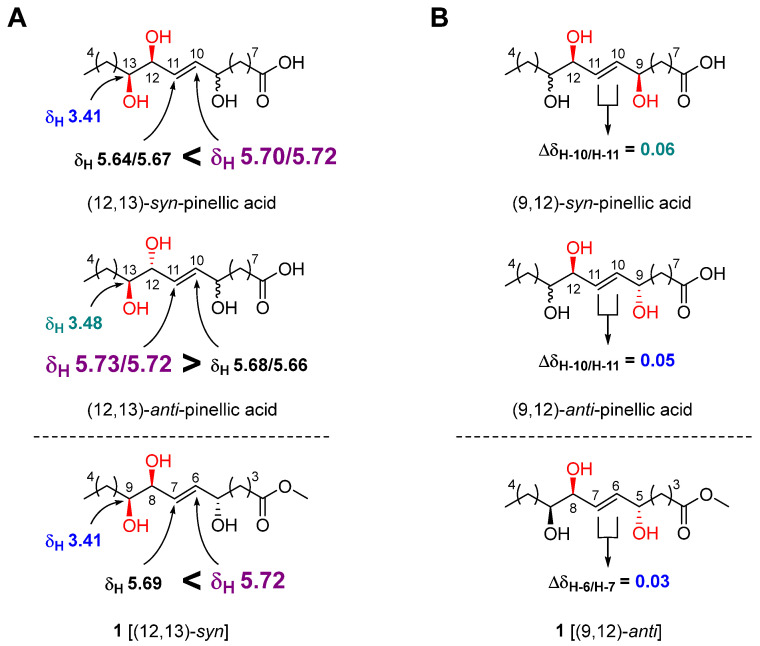
Relative stereochemical assignment of **1** at C-12/C-13 (**A**) and C-9/C-12 (**B**).

The geometry of the double bond at C-6/C-7 was determined to be *E* by observing a relatively large coupling constant of H-6/H-7 (15.5 Hz) [17]. The relative configuration of the three hydroxy groups in **1** was initially assumed to be the same as that of pinellic acid, a natural product isolated from *Pinellia ternata* [18], and further confirmed by intensive comparative ^1^H NMR data analysis as follow. In 2006, Shirahata et al. synthesized eight possible stereoisomers of pinellic acid and established ^1^H NMR (in methanol-*d*_4_)-based empirical rules for the assignment of the relative configuration of three hydroxy groups [19]. In brief, a more downfield-shifted ^1^H NMR signal of H-10 (*δ*_H_ 5.70/5.72) than of H-11 (*δ*_H_ 5.64/5.67) indicated a (12,13)-*syn* configuration, and the chemical shift of H-13 in (12,13)-*syn*-form, *δ*_H_ 3.41, was smaller than that in (12,13)-*anti*-form, *δ*_H_ 3.48 (Figure 3A). Discrimination of the (9,12)-*syn/anti* configuration was achieved by calculating the Δ*δ*_H_-_10/H-11_ value. A relatively smaller Δ*δ*_H-10/H-11_ value indicated a (9,12)-*anti* configuration [(9,12)-*syn*: 0.06 ppm, (9,12)-*anti*: 0.05 ppm]. By observing a larger *δ*_H-6_ value (5.72 ppm) than *δ*_H-7_ value (5.69 ppm), a small *δ*_H-9_ value (3.41 ppm), and a small Δ *δ*_H-6/H-7_ value (0.03 ppm) for **1**, we were able to determine the relative configuration of **1** as (5,8)-*anti* and (8,9)-*syn* (Figure 3). Therefore, the structure of **1** was elucidated as methyl (5R*,8R*,9R*)-5,8,9-trihydroxy-6*E*-tetradecenoate.

**Figure 4:**
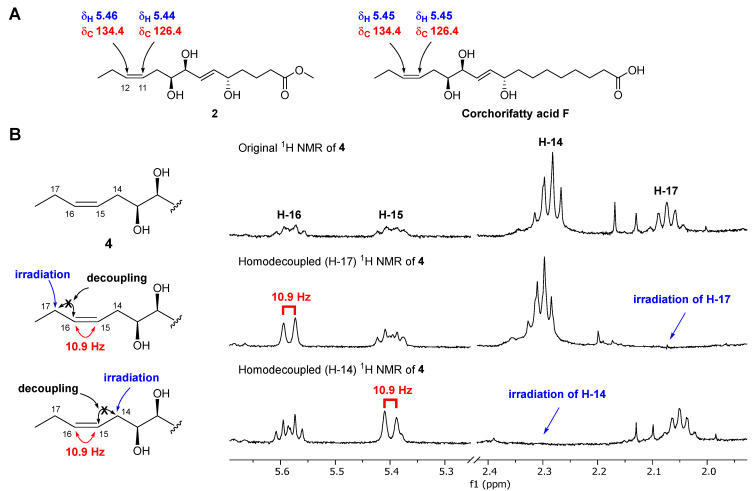
Determination of the geometry of **2** by comparative NMR data analysis measured in methanol-*d*_4_ (**A**) and of **4** by selective homonuclear decoupled ^1^H NMR experiments (**B**).

Chaenomester B (**2**) has the molecular formula C_15_H_26_O_5_, identified from the sodiated molecular ion at *m/z* 309.1672 [M + Na]^+^ (calcd. for C_15_H_26_O_5_Na^+^, *m/z* 309.1672). The ^1^H and ^13^C NMR data of **2** resembled those of **1**, except for the presence of double bond signals at *δ*_H_ 5.46 (1H, m, H-12) and 5.44 (1H, m, H-11), and *δ*_C_ 134.4 (C-12) and 126.4 (C-11) for **2** instead of two methylene signals, as observed for **1**. The location of the double bond was confirmed at C-11/C-12 by ^1^H-^1^H COSY correlations of H-10/H-11 and H-12/H-13 and HMBC correlations of H-9/C-11, H-11/C-10, H-12/C-10, H-13/C-11, H-14/C-12, and C-13 (Figure 2). The geometry of the double bond at C-11/C-12 was suggested to be *Z* based on the identical ^1^H and ^13^C NMR signals of the olefinic functionality in **2** and corchorifatty acid F, which have *Z* configuration (Figure 4A) [20]. Hence, the structure of **2** was determined as methyl (5R*,8R*,9R*)-5,8,9-trihydroxytetradeca-6*E*,11*Z*-dienoate.

The molecular formula of chaenomester C (**3**) was determined as C_20_H_38_O_5_ by observing the sodiated molecular peak at *m/z* 381.2612 [M + Na]^+^ (calcd. for C_20_H_38_O_5_Na^+^, *m/z* 381.2611) in HRFABMS. Intensive analysis of the 1D NMR data of **3** indicated that **3** would be an ethyl ester derivative of pinellic acid [18], as deduced by the additional ethyl group signals in the NMR spectra of **3** at *δ*_H_ 4.12 (2H, q, *J* = 7.1 Hz, H-19) and 1.25 (3H, t, *J* = 7.1 Hz, H-20) and *δ*_C_ 60.3 (C-19) and 14.4 (C-20). This initial proposal was supported by the 2D NMR data analysis including a HMBC correlation of H-19/C-1 and a ^1^H-^1^H COSY cross-peak of H-19/H-20 (Figure 2). Therefore, the structure of **3** was elucidated as ethyl (9R*,12R*,13R*)-9,12,13-trihydroxy-10*E*-octadecenoate.

Chaenomester D (**4**) showed the molecular formula C_20_H_36_O_5_, supported by the sodiated molecular ion peak at *m/z* 379.2455 [M + Na]^+^ (calcd. for C_20_H_36_O_5_Na^+^, *m/z* 379.2455). The NMR spectra of **4** showed that **4** is similar to **3**, with the major difference of two olefinic signals, *δ*_H_ 5.58 (1H, m, H-16) and 5.40 (1H, m, H-15) and *δ*_C_ 135.5 (C-16) and 123.9 (C-15) observed in **4**. The analysis of the ^1^H-^1^H COSY and HMBC data of **4** around the double bond led us to propose the location of the double bond at C-15/C-16 (Figure 2). We tried to measure the coupling constant between H-15 and H-16 to assign the geometry of the double bond, but we were not successful because of the complex splitting patterns of H-15 and H-16. To overcome this issue, we employed a classical selective ^1^H homonuclear decoupling technique in the ^1^H NMR measurement [21,22]. Briefly, if a proton resonance **A** is irradiated during acquisition, its coupling partner **B** loses its coupling to the irradiated proton. As a consequence, we could observe a more simplified splitting of **B** resonance and then calculate the coupling constant of **B** with other proton signal(s). As shown in the middle of Figure 4B, when H-17 was irradiated in **4**, the splitting pattern of H-16 became simplified from a multiplet to a doublet, with a coupling constant of 10.9 Hz, indicating that the geometry of the double bond at C-15/C-16 was the *cis*-form. The same result was obtained by analyzing the H-14-irradiated homonuclear decoupled ^1^H NMR spectrum (Figure 4B, bottom). Collectively, we propose the structure of **4** to be ethyl (9R*,12R*,13R*)-9,12,13-trihydroxy-10*E*-octadecenoate.

The known compound **5** was identified as 1’-hexadecanoic acid-2’,3’-dihydroxy-propylester by the comparison of its spectroscopic data with those of the reference [23].

### 3.2. Biosynthetic Proposal

**Figure 5:**
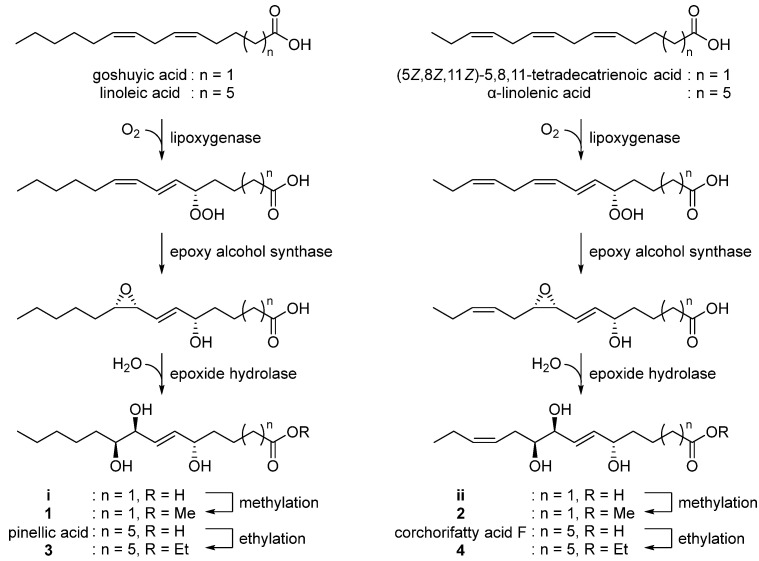
Proposed biosynthetic pathway of **1–4**.

With the characterized the structures of **1–4** in hand, we then were able to propose their biosynthetic pathway (Figure 5). Pinellic acid has been previously suggested to be synthesized from linoleic acid by lipoxygenase, epoxy alcohol synthase, and epoxide hydrolase [24]. Further ethylation on pinellic acid would produce compound **3**. Similarly, goshuyic acid, a C14 fatty acid, would be utilized to generate the acid **i**, and then compound **1** would be synthesized by methylation on **i**. We propose that compounds **2** and **4** would be produced from (5Z,8Z,11Z)-5,8,11-tetradecatrienoic acid and α-linolenic acid, respectively, by the same enzymatic reaction as those necessary for **1** and **3**.

### 3.3. Anti-Neuroinflammatory Activity of the Isolated Compounds (***1**–**5***)

**Figure 6:**
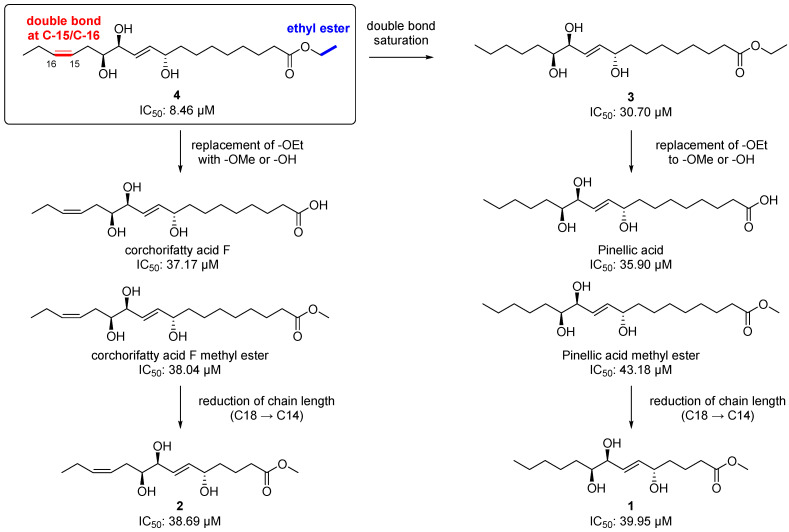
Structure–activity relationship (SARs) for the isolated metabolites (**1–4**) and other four structurally similar metabolites our group reported previously [8].

Chemically, **2** and **4** are classified as omega-3 oxylipins since they possess a double bond at the omega-3 position, and **1** and **3** are omega-6 fatty acid-derived oxylipins. Given the important roles of the omega-3 and 6 oxylipins in the inflammatory response, as we mentioned earlier, we first tested the isolated compounds (**1–5**) for their anti-inflammatory effect by measuring the level of NO production after treatment with the compounds at a concentration of 20 μM in LPS-stimulated BV-2 microglia cells. Among the tested compounds including the positive control, L-NMMA (produced NO concentration = 22.37 ± 0.69 µM), the most potent inhibitor was **4**, an omega-3 oxylipin, with an NO level of 8.46 ± 0.68 µM and without cell toxicity (Table 2). Compounds **3** and **5** also showed strong NO inhibitory activity, with reduced values of 30.70 ± 1.38 and 24.09 ± 1.11 µM, respectively, and compounds **1** (38.56 ± 1.61 μM) and **2** (38.69 ± 1.41 μM) were not active, as the NO levels were similar to that observed in the LPS-treated group (39.95 ± 1.17 μM). With these NO inhibition data for **1–5** and other four structurally similar compounds our group reported previously [8], we suggest a structure–activity relationship (SAR) for these oxylipin compounds as follow (Figure 6). The saturation of the double bond at C-15/C-16 in **4** decreased the potency about 3.8-fold [8.46 µM (**4**) → 30.70 μM (**3**)]. The replacement of an ethoxy group in **3** and **4** with a methoxy or hydroxy group reduced the activities [8.46 μM (**4**) → 37.17 and 38.04 μM (corchorifatty acid F and its methyl ester, respectively); 30.70 µM (**3**) → 35.90 and 43.18 μM (pinellic acid and its methyl ester, respectively)]. There was no significant change in the IC_50_ values when the chain length was reduced from 18-carbon [corchorifatty acid F methyl ester, 38.04 μM; pinellic acid methyl ester, 43.18)] to 14-carbon [**2**, 38.69 μM; **1**, 38.56 μM)]. Collectively, we propose that the presence of a double bond at C-15/C-16 (omega-3 position) and of an ethyl ester functionality is important for a strong anti-inflammatory activity, which is consistent with the fact that some omega-3 oxylipins such as EPA and DHA are anti-inflammatory agents [2,25]. In addition, an ethyl ester is generally more than two-fold stable than a methyl ester during hydrolysis, indicating that an ethyl ester would lend not only anti-neuroinflammatory activity but also stability to the molecule [26].

### 3.4. Neurotrophic Activity of the Isolated Compounds (***1**–**5***)

In addition to the anti-neuroinflammatory effect, several omega-3 oxylipins have also shown neuroprotective effect by decreasing inflammation and apoptosis [27,28,29], inhibiting astrogliosis [30], activating mitochondria [29], promoting angiogenesis, and regulating the blood flow [30,31]. Therefore, we also evaluated the compounds **1**–**5** for their neurotrophic effects and ability to induce NGF secretion from C6 glioma cells (Table 3). Compound **4** exhibited a significant NGF-stimulating effect, inducing NGF secretion that was 157.7 ± 2.4% (^***^p < 0.001) of that measured for the untreated control, without cell toxicity, and stronger than that induced by 6-shogaol (154.0 ± 5.6%, ^**^p < 0.01), the positive control. Compared with the untreated control group, compounds **3** and **5** were mild NGF inducers, with 126.7 ± 2.4% (^*^p < 0.05) and 123.8 ± 11.0% (^*^p < 0.05) of stimulating potency, respectively, and compounds **1** and **2** were inactive (NGF secretion ≈100%).

### 3.5. Cytotoxic Activity of the Isolated Compounds (***1**–**5***)

Lastly, the cytotoxic activity of the isolates (**1–5**) was examined using four human tumor cell lines (A549, SK-OV-3, SK-MEL-2, and MKN-1) by the SRB assay. As shown in Table 4, compound **4** inhibited the proliferation of A549 cell, showing an IC_50_ value of 27.4 μM, while **5** was cytotoxic in all tested cell lines (IC_50_ 15.1–26.6 μM). Compounds **1–3** were not active (IC_50_ >30 μM). Etoposide was used as a positive control (IC_50_ 1.0–3.5 μM). 

## 4. Conclusions

Our current study revealed four new oxylipins, chaenomesters A–D (**1–4**), isolated from the twigs of *C. sinensis* along with the structurally similar known compound **5**. The structural elucidation of the isolates was performed mainly by HRMS and NMR data analysis, and the stereochemical assignments were achieved by ^1^H NMR-based empirical rules and homonuclear decoupled ^1^H NMR experiments. Among the isolates, chaenomester D (**4**), an omega-3 oxylipin, showed potent neurotrophic activity in C6 glioma cells, mild anti-neuroinflammatory activity in LPS-stimulated BV-2 microglia cells, and mild cytotoxic activity in A549 cancer cells. The SAR study suggested the double bond at C-15/C-16 and the ethyl ester functionality in chaenomester D (**4**) would play an important role in the production of a potent anti-inflammatory effect. Collectively, we propose that chaenomester D (**4**) could contribute to the development of new drugs to treat neurodegenerative diseases such as Alzheimer’s disease and Parkinson’s disease.

## Figures and Tables

**Table 2:** Inhibitory effects on NO production of compounds **1–5** at 20 μM in LPS-stimulated BV-2 cells.

Compound	Normalized NO production (µM) ^1^	Cell viability (%) ^2^
**DMSO**	2.33 ± 0.58	117.52 ± 1.04
**LPS-treated**	39.95 ± 1.17 ^###^	100.00 ± 0.88
**1**	38.56 ± 1.61	105.36 ± 3.55
**2**	38.69 ± 1.41	97.39 ± 7.89
**3**	30.70 ± 1.38 ***	121.17 ± 8.80
**4**	8.46 ± 0.68 ***	97.77 ± 1.23
**5**	24.09 ± 1.11 ***	109.15 ± 1.81
L-NMMA ^3^	22.37 ± 0.69 ***	104.56 ± 4.21

^1 ^The NO levels produced by the cells is expressed as µM and determined by using the standard curve of sodium nitrite. The data shown represent the means ± SD of three independent experiments performed in triplicate. Significant differences between experimental groups were determined using one-way analysis of variance followed by Tukey’s post-hoc test to measure statistical significance using GraphPad Prism 8. Statistical significance was set to ^###^ *p* < 0.001 vs. DMSO control group, *** *p* < 0.001 vs. LPS-treated control group. ^2 ^Cell viability after treatment with each compound at 20 µM concentration was determined by the MTT assay and is expressed in percentage (%). ^3 ^L-NMMA was used as a positive control.

**Table 3:** Effects of the isolated compounds (**1–5**) on nerve growth factor (NGF) secretion in C6 cells.

Compound	Normalized NGF secretion (%) ^1^	Cell viability (%) ^2^
**DMSO**	100.0 ± 0.6	100.0 ± 1.1
**1**	79.6 ± 3.4	98.5 ± 0.8
**2**	86.0 ± 4.4	109.9 ± 9.3
**3**	126.7 ± 2.4 *	105.3 ± 1.6
**4**	157.7 ± 2.4 ***	107.7 ± 11.2
**5**	123.8 ± 11.0 *	101.6 ± 1.4
6-shogaol ^3^	154.0 ± 5.6 **	97.1 ± 0.2

^1 ^C6 cells were treated with the compounds at 20 µM concentration. After 24 h, the level of NGF secretion in C6-conditioned medium was measured by ELISA. The level of secreted NGF from the cells is expressed as a percentage of the level measured for the untreated control divided by the respective cell viability values. The data shown represent the means ± SD of three independent experiments performed in triplicate. Significant differences between the experimental groups were determined using one-way analysis of variance followed by the Newman–Keuls post hoc test, used to measure statistical significance using GraphPad Prism 8. Statistical significance was set to * *p* < 0.05, ** *p* < 0.01, and *** *p* < 0.001 vs. control group ^2 ^Cell viability after treatment with each compound at 20 µM concentration was determined by the MTT assay and is expressed in percentage (%). The results are averages of three independent experiments, and the data are expressed as mean ± SD; ^3 ^6-shogaol was the positive control.

**Table 4:** Cytotoxicity of compounds **4** and **5** in four cultured human cancer cell lines measured with the SRB bioassay.

Compound	IC_50_ (μM) ^1^			
	A549	SK-OV-3	SK-MEL-2	MKN-1
**1**	>30	>30	>30	>30
**2**	>30	>30	>30	>30
**3**	>30	>30	>30	>30
**4**	27.4	>30	>30	>30
**5**	17.5	18.9	15.1	26.6
Etoposide ^2^	1.0	2.2	1.8	3.5

^1 ^The IC_50_ value of each compound is defined as the concentration (μM) that caused a 50% inhibition in cell proliferation; a compound is considered inactive when its IC_50_ is over 30 µM. ^2 ^Positive control.

## Data Availability

The data presented in this study are available on request from the corresponding author.

## Supplementary Materials

The following are available online at https://www.mdpi.com/article/10.3390/antiox12020284/s1, Figures S1–S24: HRFABMS ^1^H, ^13^C, COSY, HSQC, and HMBC NMR spectra of **1–4**.

## References

[1] Gabbs M., Leng S., Devassy J.G., Monirujjaman M., Aukema H.M. (2015). Advances in our understanding of oxylipins derived from dietary PUFAs. Adv. Nutr..

[2] Spite M., Clària J., Serhan C.N. (2014). Resolvins, specialized proresolving lipid mediators, and their potential roles in metabolic diseases. Cell Metab..

[3] Wongrakpanich S., Wongrakpanich A., Melhado K., Rangaswami J. (2018). A comprehensive review of non-steroidal anti-inflammatory drug use in the elderly. Aging Dis..

[4] Cho C.K., Park H.-J., Kang P., Moon S., Lee Y.J., Bae J.W., Jang C.-G., Lee S.-Y. (2021). Physiologically based pharmacokinetic (PBPK) modeling of meloxicam in different CYP2C9 genotypes. Arch. Pharm. Res..

[5] Kim Y.-H., Kang P., Cho C.K., Jung E.H., Park H.-J., Lee Y.J., Bae J.W., Jang C.-G., Lee S.-Y. (2021). Physiologically based pharmacokinetic (PBPK) modeling for prediction of celecoxib pharmacokinetics according to CYP2C9 genetic polymorphism. Arch. Pharm. Res..

[6] Cho C.K., Kang P., Park H.-J., Ko E., Mu C.Y., Lee Y.J., Choi C.-I., Kim H.S., Jang C.-G., Bae J.W. (2022). Physiologically based pharmacokinetic (PBPK) modeling of piroxicam with regard to CYP2C9 genetic polymorphism. Arch. Pharm. Res..

[7] Delker C., Stenzel I., Hause B., Miersch O., Feussner I., Wasternack C. (2006). Jasmonate biosynthesis in *Arabidopsis thaliana*-enzymes, products, regulation. Plant Biol..

[8] Kim C.S., Kwon O.W., Kim S.Y., Choi S.U., Kim K.H., Lee K.R. (2014). Five new oxylipins from *Chaenomeles sinensis*. Lipids.

[9] Park J.E., Lee T.H., Ham S.L., Subedi L., Hong S.M., Kim S.Y., Choi S.U., Kim C.S., Lee K.R. (2022). Anticancer and anti-neuroinflammatory constituents isolated from the roots of *Wasabia japonica*. Antioxidants.

[10] Kim C.S., Suh W.S., Subedi L., Kim S.Y., Choi S.U., Lee K.R. (2016). Neuroprotective fatty acids from the stem bark of *Sorbus commixta*. Lipids.

[11] Kim C.S., Kim K.H., Lee K.R. (2014). Phytochemical constituents of the leaves of *Hosta longipes*. Nat. Prod. Sci..

[12] Kim D.H., Subedi L., Kim H.R., Choi S.U., Kim S.Y., Kim C.S. (2021). Phenolic constituents of chinese quince twigs (*Chaenomeles sinensis Koehne*) and their anti-neuroinflammatory, neurotrophic, and cytotoxic activities. Antioxidants.

[13] Blasi E., Barluzzi R., Bocchini V., Mazzolla R., Bistoni E.F. (1990). Immortalization of murine microglial cells by a v-raf/v-myc carrying retrovirus. J. Neuroimmunol..

[14] Kim J.M., Son D., Lee P., Lee K.J., Kim H., Kim S.Y. (2003). Ethyl acetate soluble fraction of cnidium officinale MAKINO Inhibits neuronal cell death by reduction of excessive nitric oxide production in lipopolysaccharide-treated rat hippocampal slice culturesand microglia cells. J. Pharmacol. Sci..

[15] Skehan P., Storeng R., Scudiero D., Monks A., McMahon J., Vistica D., Warren J.T., Bokesch H., Kenney S., Boyd M.R. (1990). New colorimetric cytotoxicity assay for anticancer-drug screening. J. Natl. Cancer Inst..

[16] Miura A., Kuwahara S. (2009). A concise synthesis of pinellic acid using a cross-metathesis approach. Tetrahedron.

[17] Zhang J.-S., Xu D.-F., Wang Y.-Y., Ma R.-F., Zhang H. (2022). Clerodane furanoditerpenoids from the stems of *Tinospora sinensis*. Arch. Pharm. Res..

[18] Nagai T., Kiyohara H., Munakata K., Shirahata T., Sunazuka T., Harigaya Y., Yamada H. (2002). Pinellic acid from the tuber of *Pinellia ternata* Breitenbach as an effective oral adjuvant for nasal influenza vaccine. Int. Immunopharmacol..

[19] Shirahata T., Sunazuka T., Yoshida K., Yamamoto D., Harigaya Y., Kuwajima I., Nagai T., Kiyohara H., Yamada H., Ōmura S. (2006). Total synthesis, elucidation of absolute stereochemistry, and adjuvant activity of trihydroxy fatty acids. Tetrahedron.

[20] Yoshikawa M., Murakami T., Shimada H., Yoshizumi S., Saka M., Yamahara J., Matsuda H. (1998). Medicinal foodstuffs. XIV. On the bioactive constituents of moroheiya.(2): New fatty acids, corchorifatty acids A, B, C, D, E, and F, from the leaves of *Corchorus olitorius L.*(Tiliaceae): Structures and inhibitory effect on NO production in mouse peritoneal macrophages. Chem. Pharm. Bull..

[21] Chang C.-H., Lee Y.-C., Hsiao G., Chang L.-K., Chi W.-C., Cheng Y.-C., Huang S.-J., Wang T.-C., Lu Y.-S., Lee T.-H. (2022). Anti-epstein–barr viral agents from the medicinal herb-derived fungus *Alternaria alstroemeriae* Km2286. J. Nat. Prod..

[22] Stierle A.A., Stierle D.B., Decato D., Alverson J., Apedaile L. (2021). Cryptic biosynthesis of the berkeleypenostatins from coculture of extremophilic *Penicillium* sp.. J. Nat. Prod..

[23] Kim C., Ha H., Kim J.S., Kim Y.T., Kwon S.-C., Park S.W. (2003). Induction of growth hormone by the roots of *Astragalus membranaceus* in pituitary cell culture. Arch. Pharm. Res..

[24] Hamberg M., Olsson U. (2011). Efficient and specific conversion of 9-lipoxygenase hydroperoxides in the beetroot. Formation of pinellic acid. Lipids.

[25] Serhan C.N., Chiang N., Van Dyke T.E. (2008). Resolving inflammation: Dual anti-inflammatory and pro-resolution lipid mediators. Nat. Rev. Immunol..

[26] Hay R., Porter L., Morris P. (1966). The basic hydrolysis of amino acid esters. Aust. J. Chem..

[27] Bazan N.G., Eady T.N., Khoutorova L., Atkins K.D., Hong S., Lu Y., Zhang C., Jun B., Obenaus A., Fredman G. (2012). Novel aspirin-triggered neuroprotectin D1 attenuates cerebral ischemic injury after experimental stroke. Exp. Neurol..

[28] Marcheselli V.L., Hong S., Lukiw W.J., Tian X.H., Gronert K., Musto A., Hardy M., Gimenez J.M., Chiang N., Serhan C.N. (2003). Novel docosanoids inhibit brain ischemia-reperfusion-mediated leukocyte infiltration and pro-inflammatory gene expression. J. Biol. Chem..

[29] Wang L., Chen M., Yuan L., Xiang Y., Zheng R., Zhu S. (2014). 14, 15-EET promotes mitochondrial biogenesis and protects cortical neurons against oxygen/glucose deprivation-induced apoptosis. Biochem. Biophys. Res. Commun..

[30] Liu Y., Wan Y., Fang Y., Yao E., Xu S., Ning Q., Zhang G., Wang W., Huang X., Xie M. (2016). Epoxyeicosanoid signaling provides multi-target protective effects on neurovascular unit in rats after focal ischemia. J. Mol. Neurosci..

[31] Zhang W., Otsuka T., Sugo N., Ardeshiri A., Alhadid Y.K., Iliff J.J., DeBarber A.E., Koop D.R., Alkayed N.J. (2008). Soluble epoxide hydrolase gene deletion is protective against experimental cerebral ischemia. Stroke.

