# Supplementary material for: Anti-Inflammatory, Neurotrophic, and Cytotoxic Oxylipins Isolated from Chaenomeles sinensis Twigs
Source: Antioxidants (Basel). 2023 Jan 27;12(2):284. doi: 10.3390/antiox12020284 (PMC9952087; doi:10.3390/antiox12020284)

Anti-inflammatory, Neurotrophic, and Cytotoxic Oxylipins Isolated from *Chaenomeles sinensis* Twigs

Da Yeong Lee,[1,#] Kyoung Jin Park,[2,#] Subedi Lalita,[3] Gyusung Lee,[1] Ji-Hyeok Lee,[4] Won-Min Lee,[1] Sang Un Choi,[5] Seong-Min Hong,[6] Sun Yeou Kim,[3,6] Chung Sub Kim[1,2,4,*]

[1]Department of Biopharmaceutical Convergence, Sungkyunkwan University, Suwon 16419, Republic of Korea

[2]School of Pharmacy, Sungkyunkwan University, Suwon 16419, Republic of Korea

[3]Gachon Institute of Pharmaceutical Science, Gachon University, Incheon 21936, Korea

[4]Department of Biohealth Regulatory Science, Sungkyunkwan University, Suwon 16419, Republic of Korea

[5]Korea Research Institute of Chemical Technology, Daejeon 34114, Korea

[6]College of Pharmacy, Gachon University, #191, Hambakmoero, Yeonsu-gu, Incheon 21936, Korea

[#]These authors contributed equally to this work.

Contents

Figure S1. HRFABMS spectrum of **1**........1

Figure S2. ^{1}H NMR spectrum of **1** in methanol-d_4 (700 MHz)........2

Figure S3. ^{13}C NMR spectrum of **1** in methanol-d_4 (175 MHz)........3

Figure S4. COSY spectrum of **1** in methanol-d_4........4

Figure S5. HSQC spectrum of **1** in methanol-d_4........5

Figure S6. HMBC spectrum of **1** in methanol-d_4........6

Figure S7. HRFABMS spectrum of **2**........7

Figure S8. ^{1}H NMR spectrum of **2** in methanol-d_4 (700 MHz)........8

Figure S9. ^{13}C NMR spectrum of **2** in methanol-d_4 (175 MHz)........9

Figure S10. COSY spectrum of **2** in methanol-d_4........10

Figure S11. HSQC spectrum of **2** in methanol-d_4........11

Figure S12. HMBC spectrum of **2** in methanol-d_4........12

Figure S13. HRFABMS spectrum of **3**........13

Figure S14. ^{1}H NMR spectrum of **3** in chloroform-*d* (700 MHz)........14

Figure S15. ^{13}C NMR spectrum of **3** in chloroform-*d* (175 MHz)........15

Figure S16. COSY spectrum of **3** in chloroform-*d*........16

Figure S17. HSQC spectrum of **3** in chloroform-*d*........17

Figure S18. HMBC spectrum of **3** in chloroform-*d*........18

Figure S19. HRFABMS spectrum of **4**........19

Figure S20. ^{1}H NMR spectrum of **4** in chloroform-*d* (700 MHz)........20

Figure S21. ^{13}C NMR spectrum of **4** in chloroform-*d* (175 MHz)........21

Figure S22. COSY spectrum of **4** in chloroform-*d*........22

Figure S23. HSQC spectrum of **4** in chloroform-*d*........23

Figure S24. HMBC spectrum of **4** in chloroform-*d*........24

Figure S1. HRFABMS spectrum of **1**

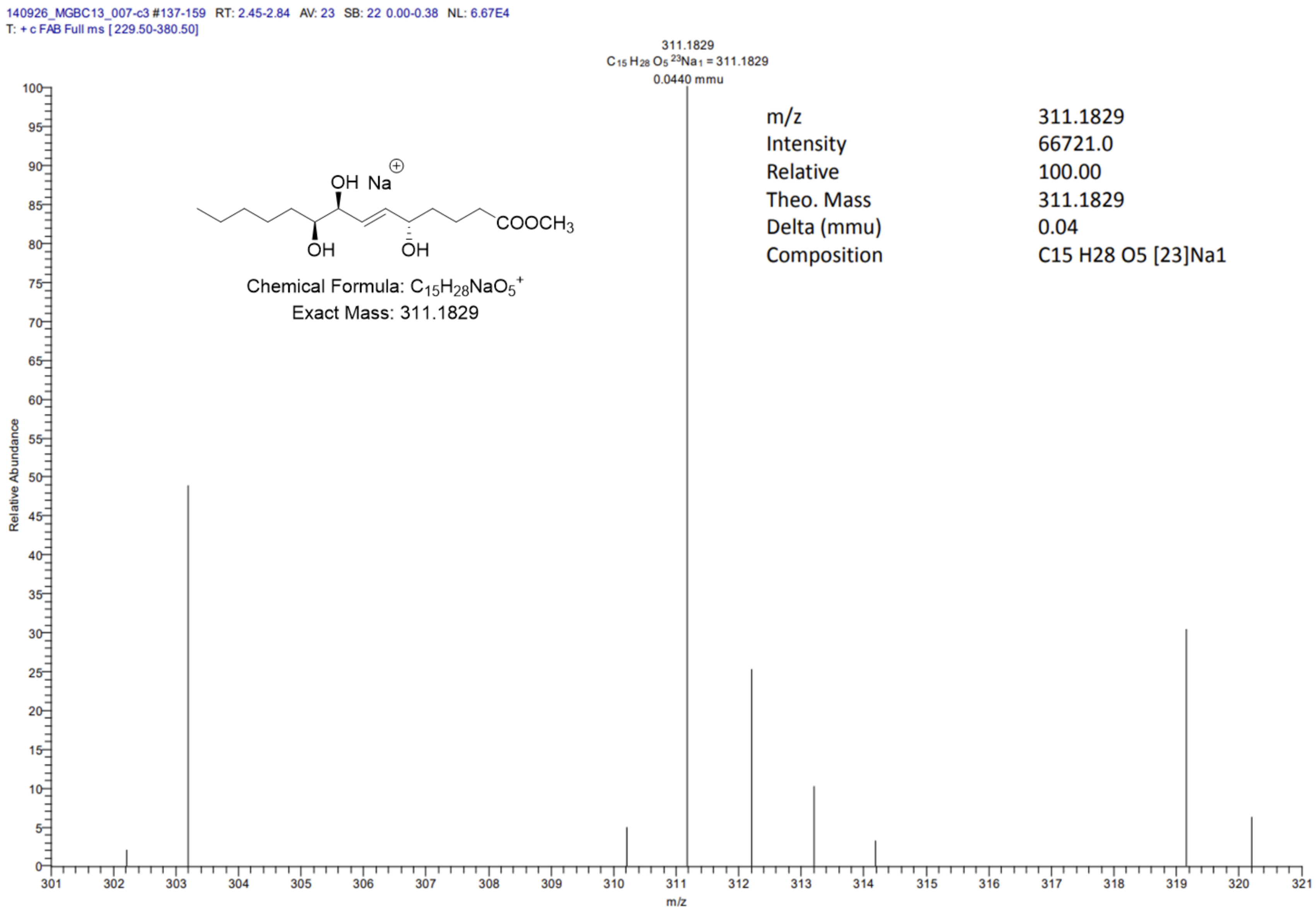

Figure S2. ^{1}H NMR spectrum of **1** in methanol-d_4 (700 MHz)

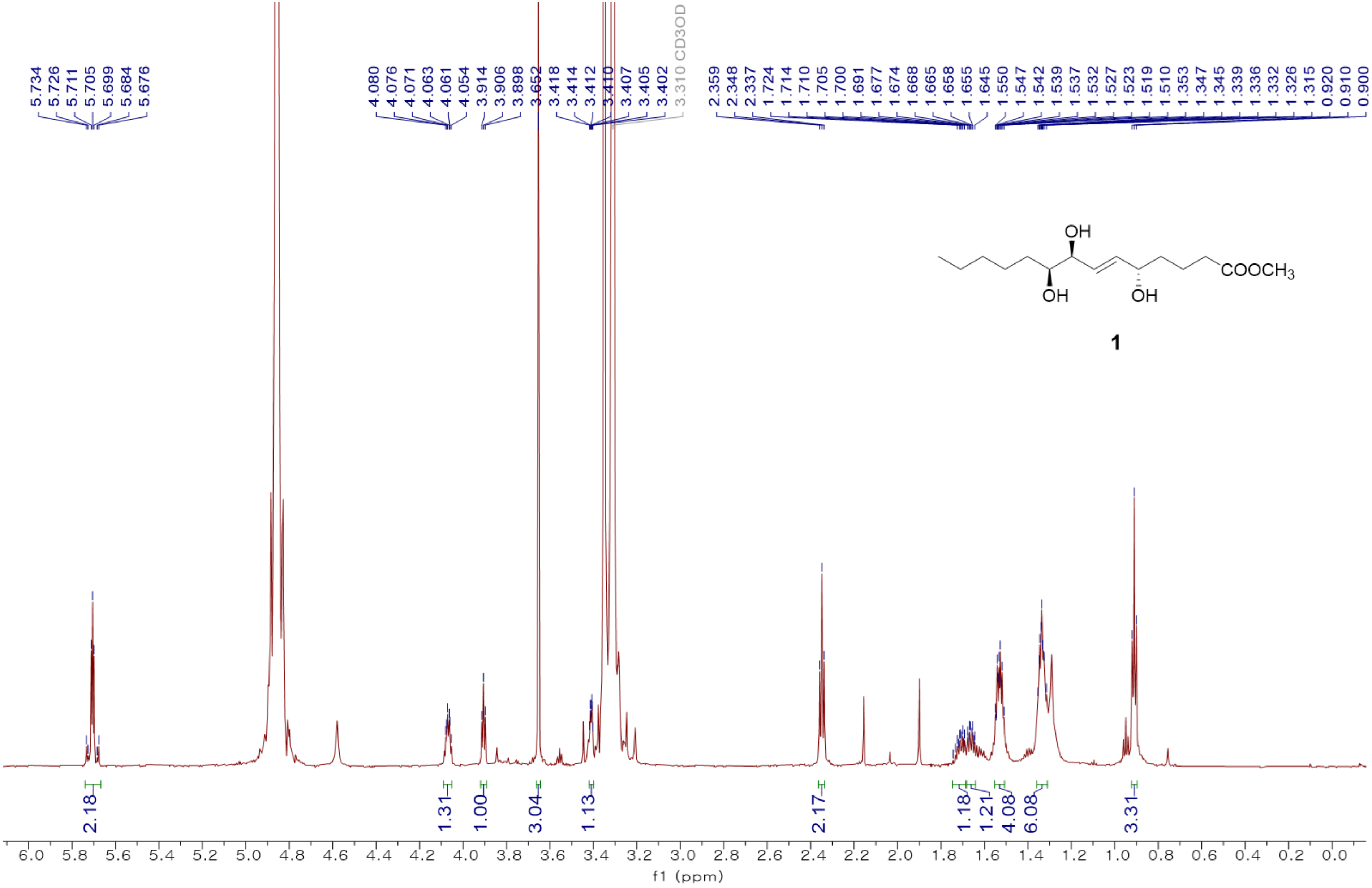

Figure S3. ^{13}C NMR spectrum of **1** in methanol-d_4 (175 MHz)

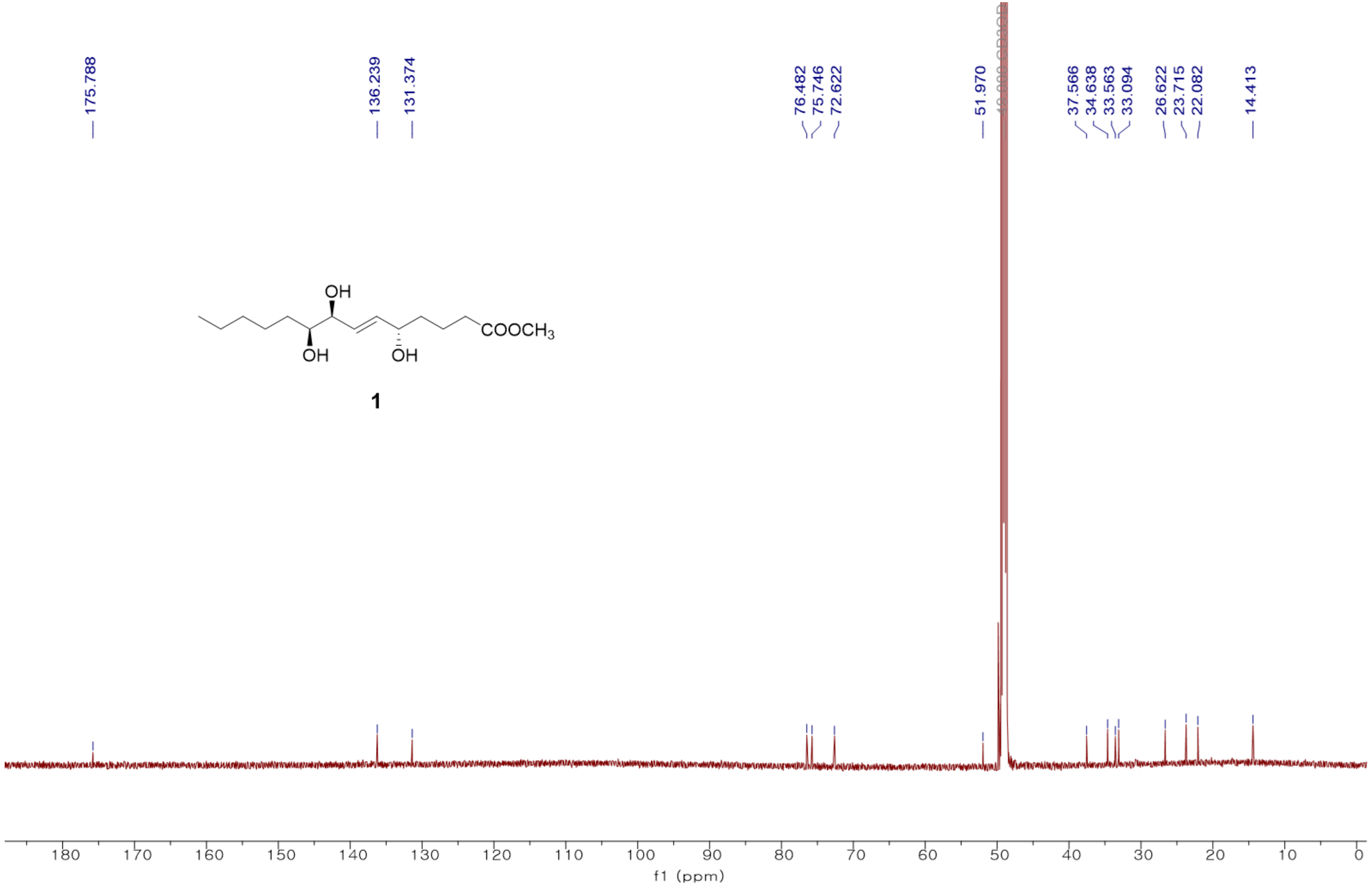

Figure S4. COSY spectrum of **1** in methanol-d_4

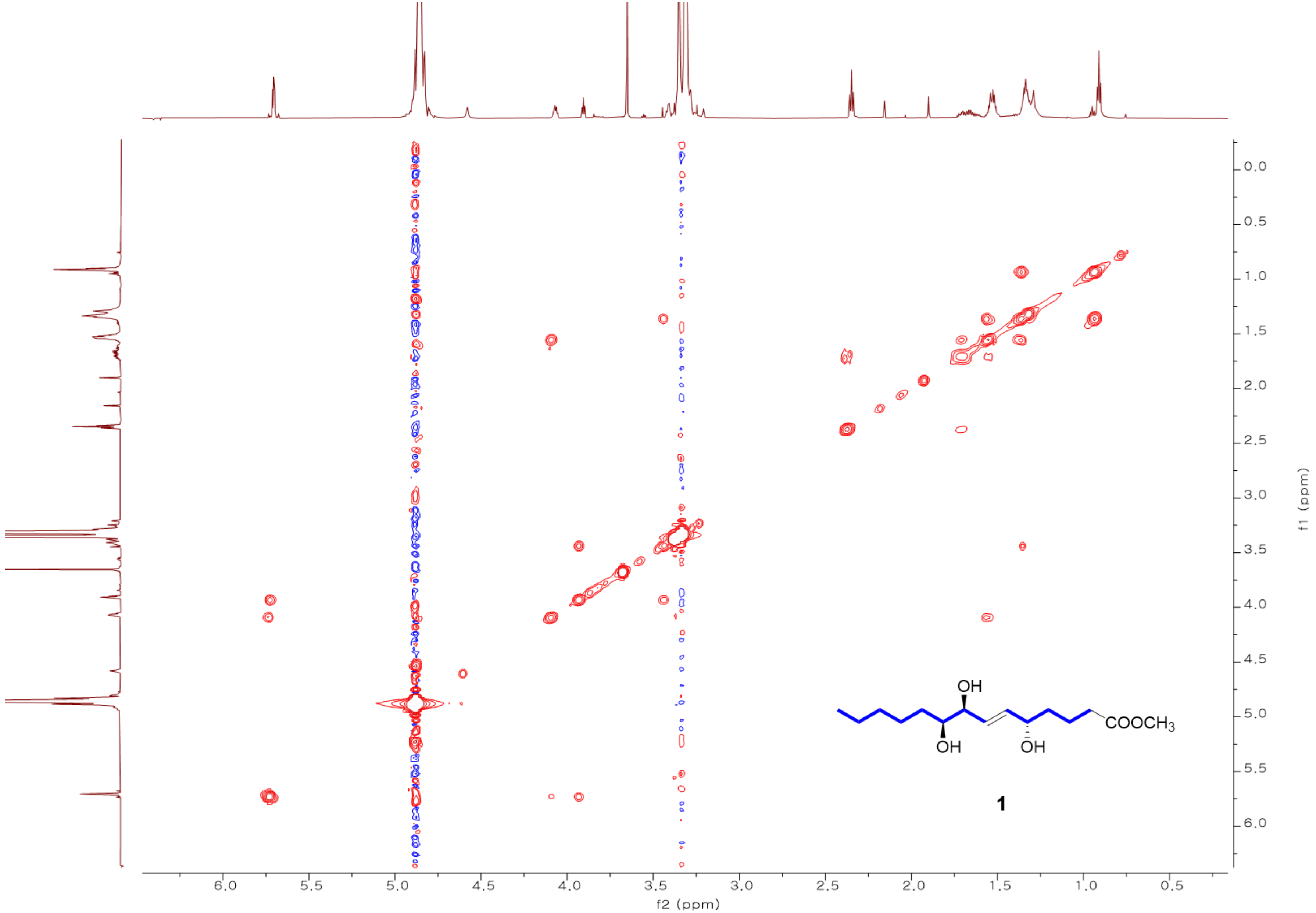

Figure S5. HSQC spectrum of **1** in methanol-d_4

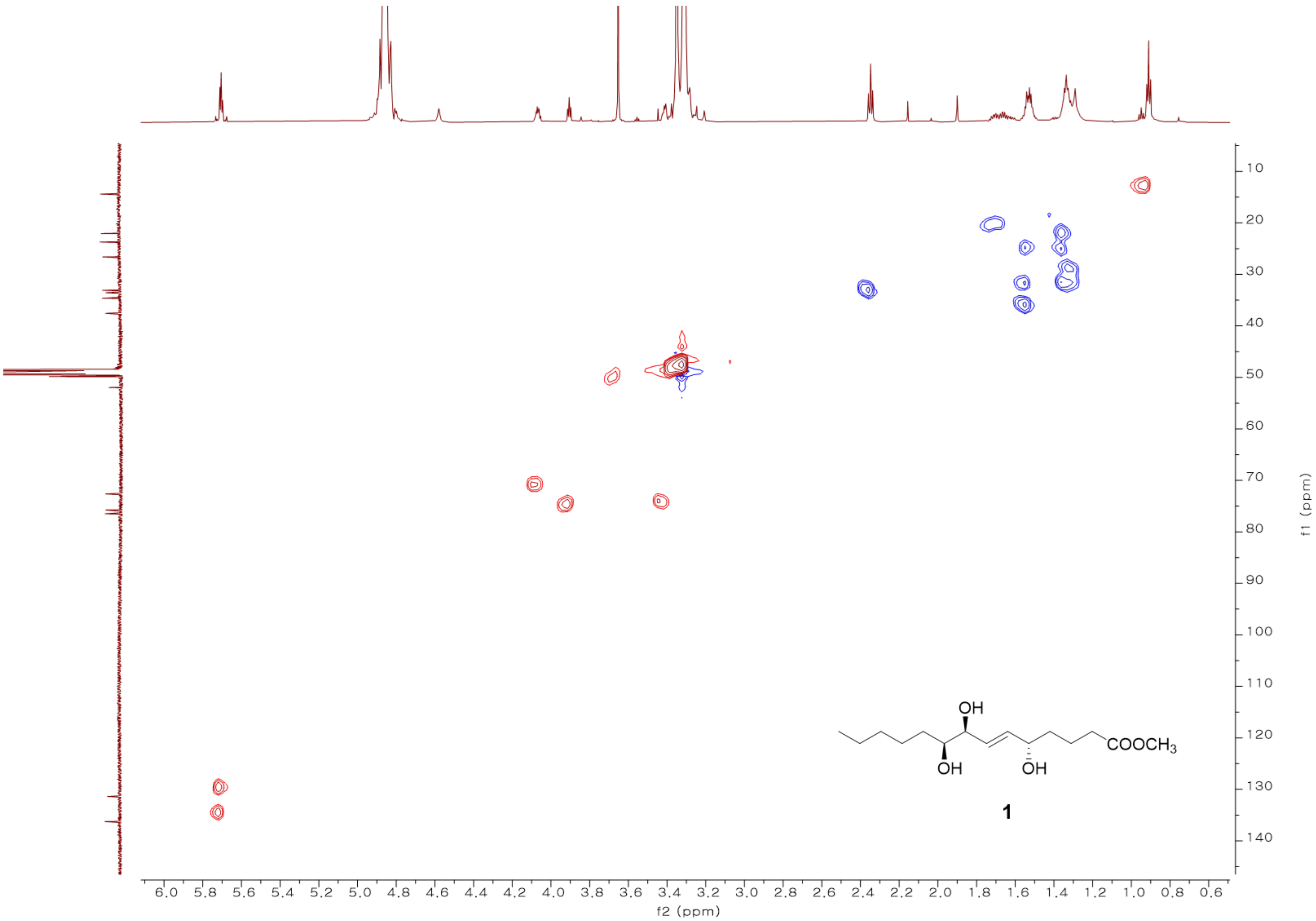

Figure S6. HMBC spectrum of **1** in methanol-d_4

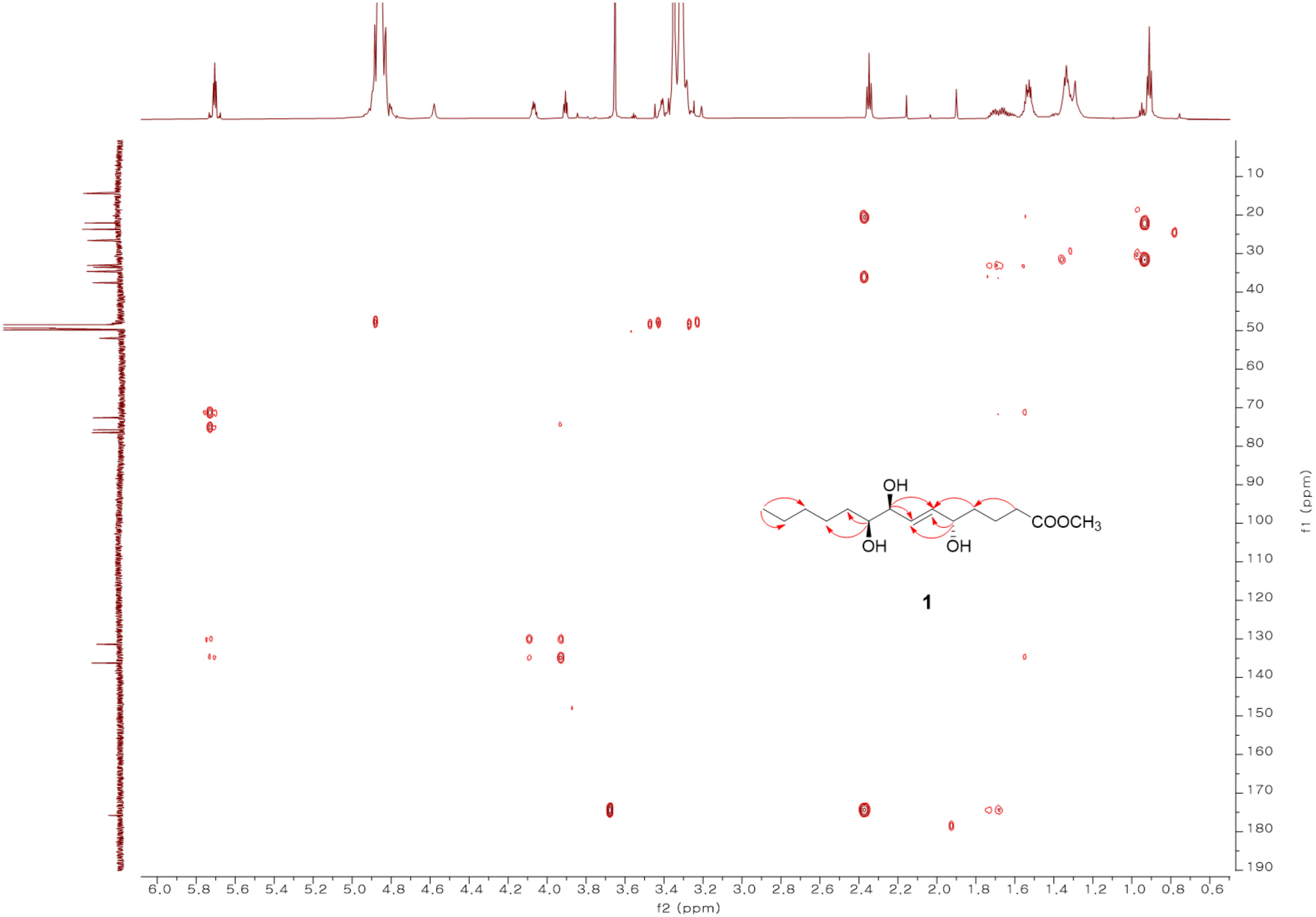

Figure S7. HRFABMS spectrum of **2**

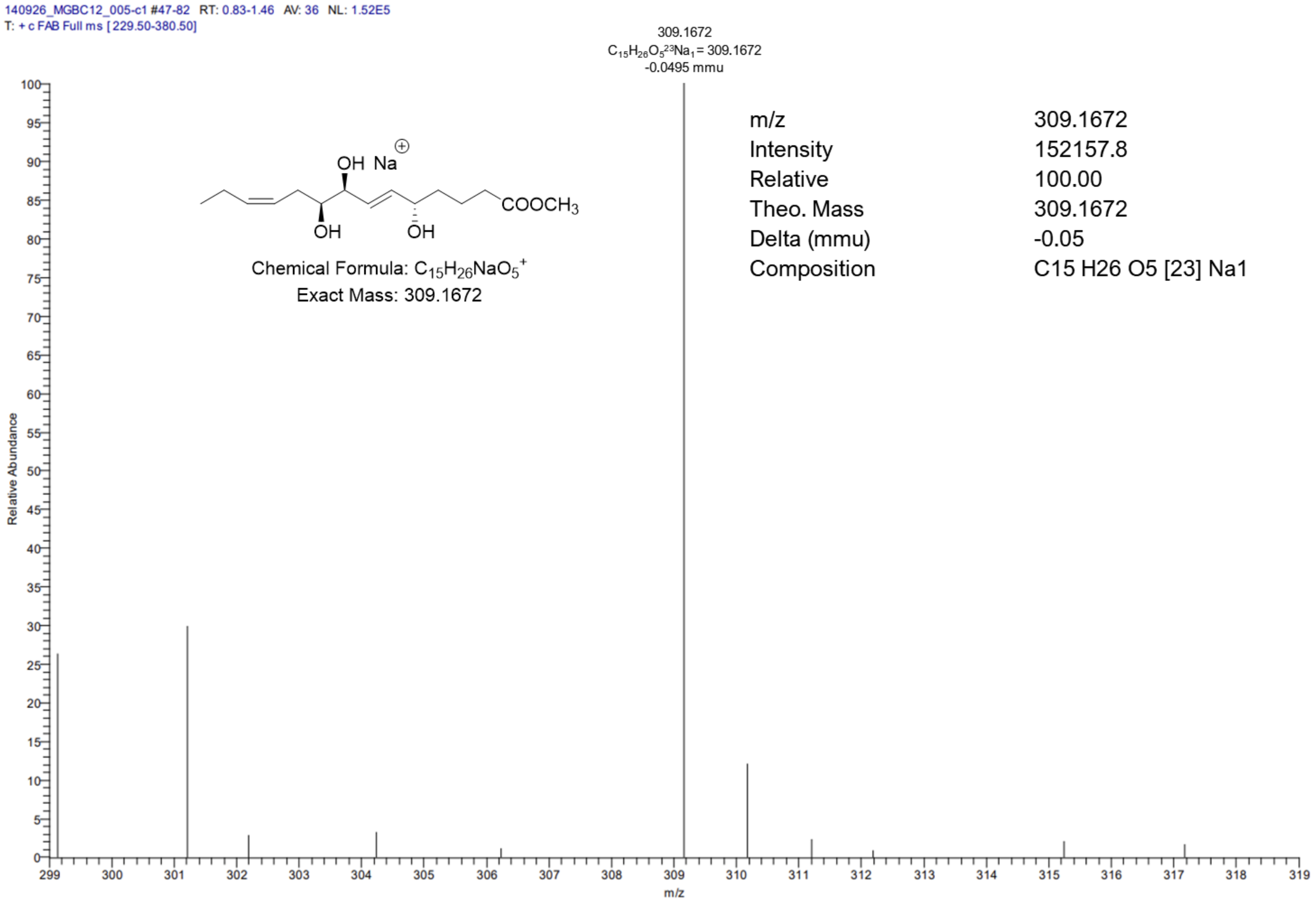

Figure S8. ^{1}H NMR spectrum of **2** in methanol-d_4 (700 MHz)

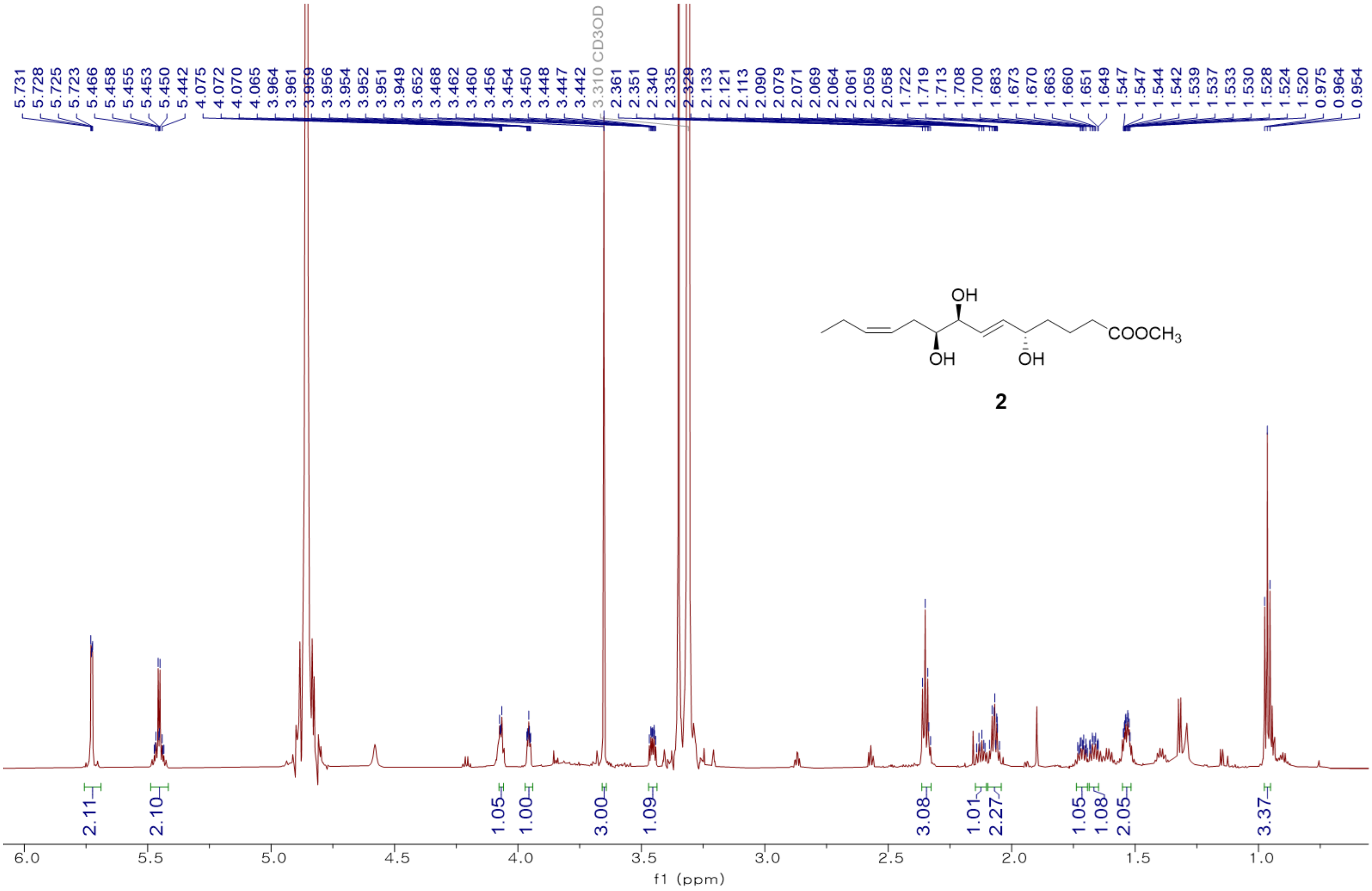

Figure S9. ^{13}C NMR spectrum of **2** in methanol-d_4 (175 MHz)

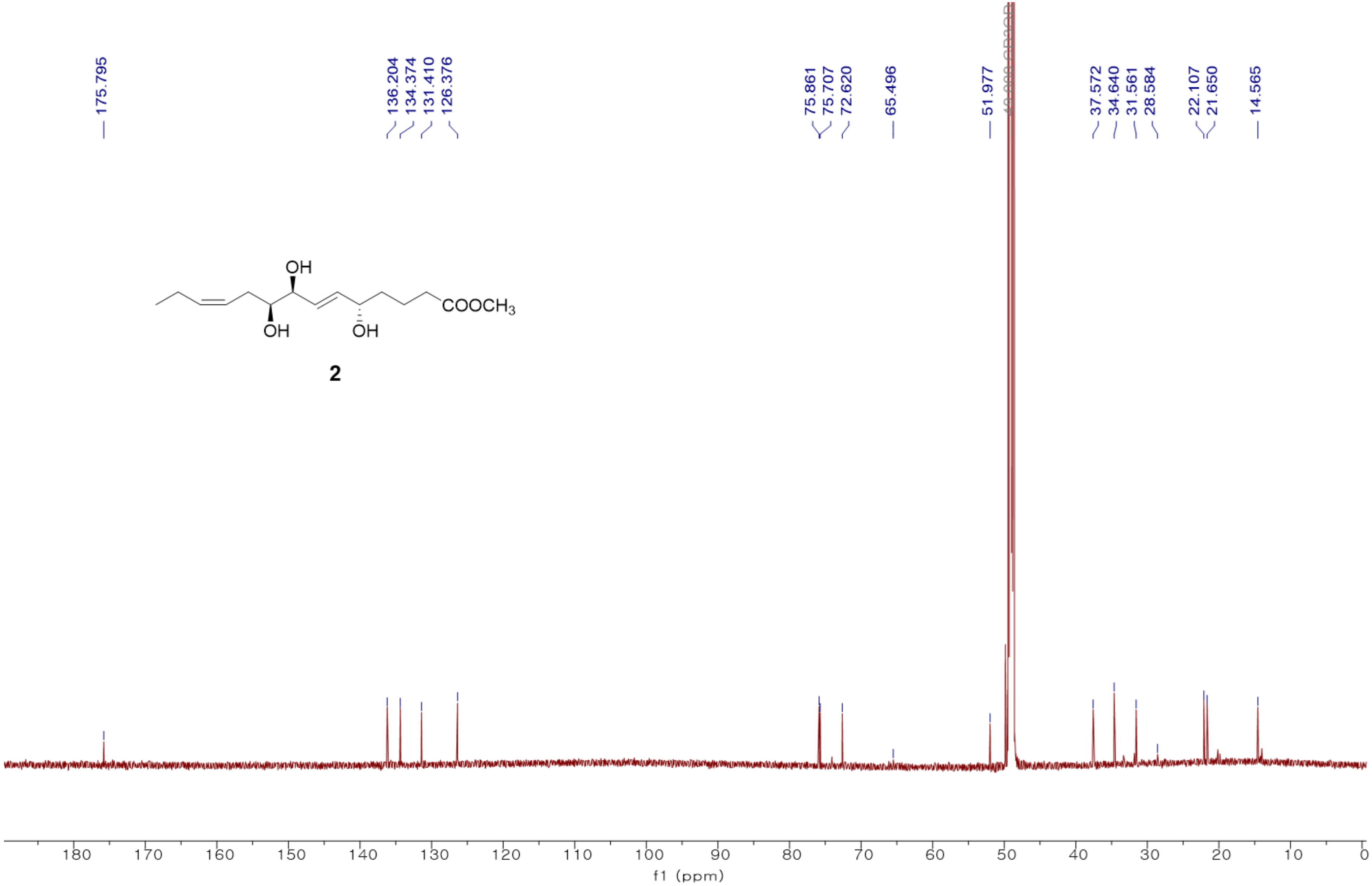

Figure S10. COSY spectrum of **2** in methanol-d_4

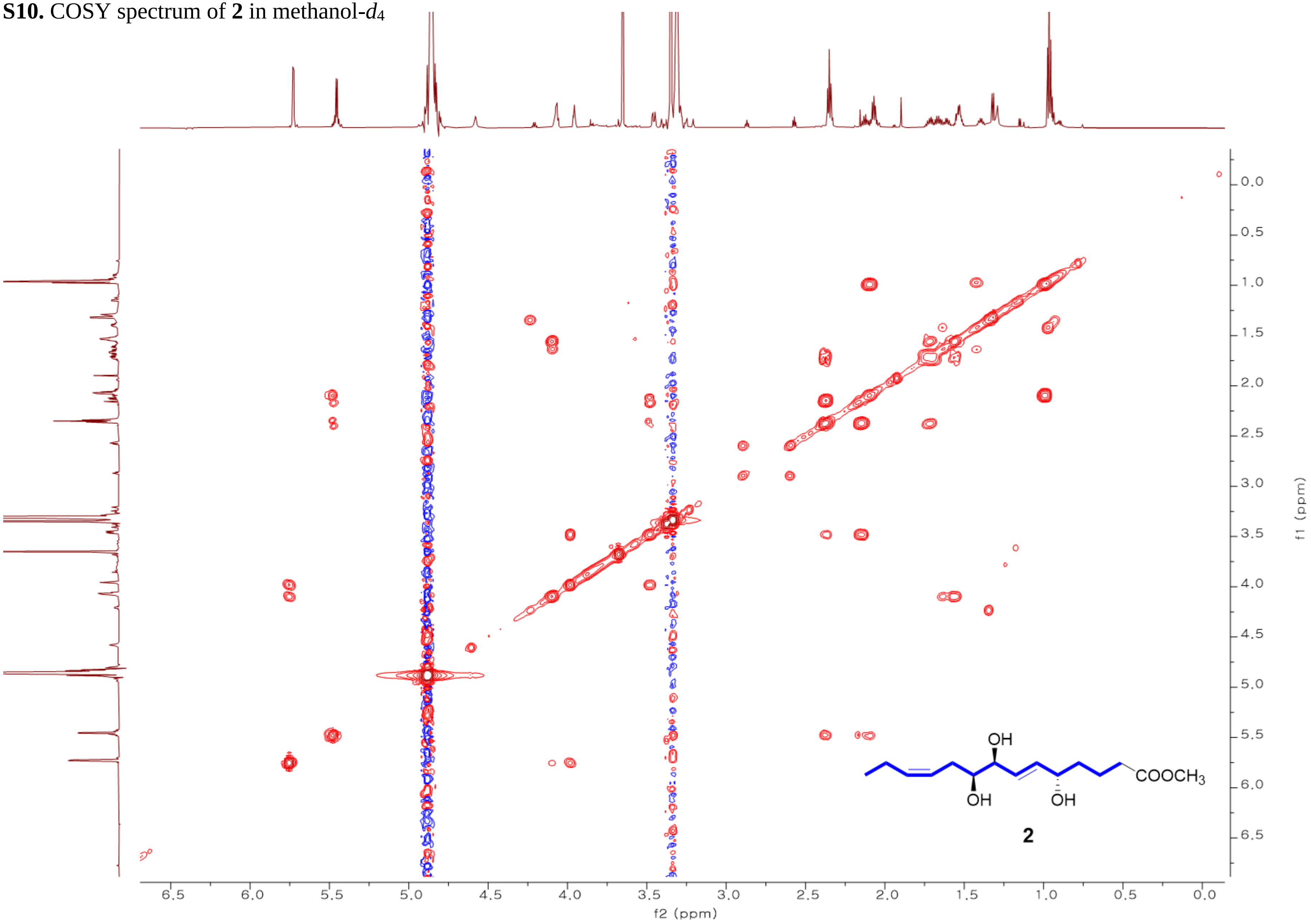

Figure S11. HSQC spectrum of **2** in methanol-d_4

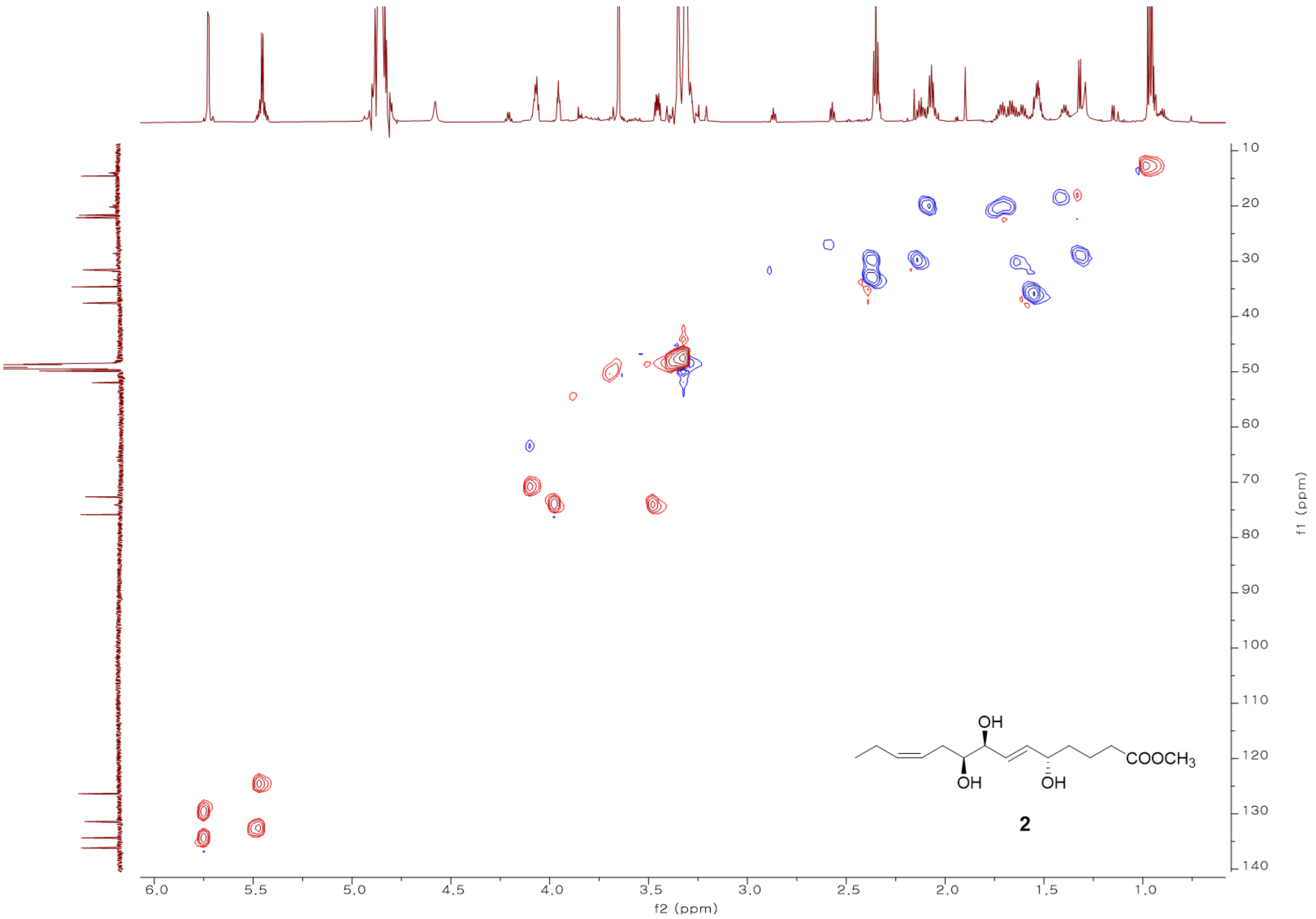

Figure S12. HMBC spectrum of **2** in methanol-d_4

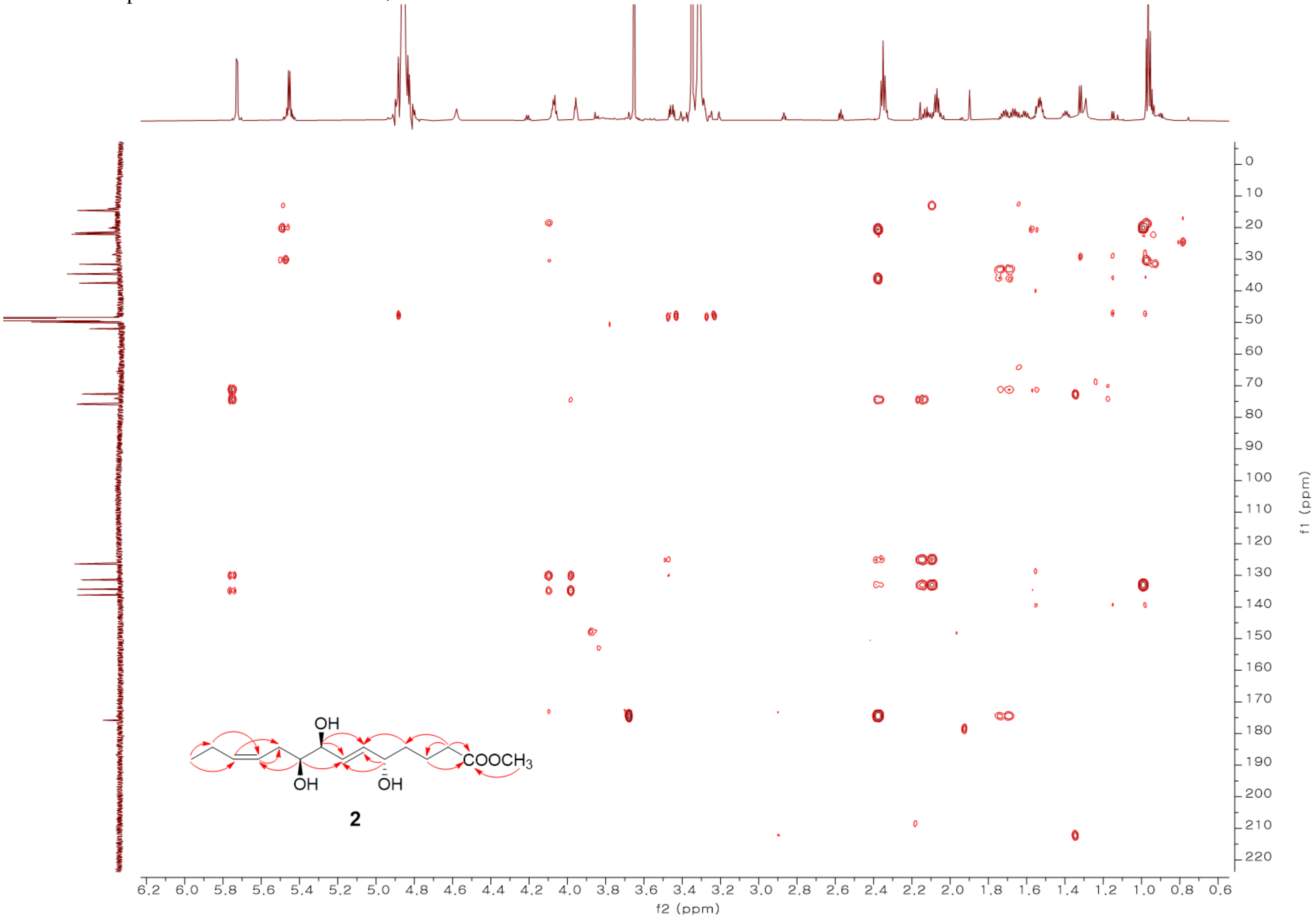

Figure S13. HRFABMS spectrum of **3**

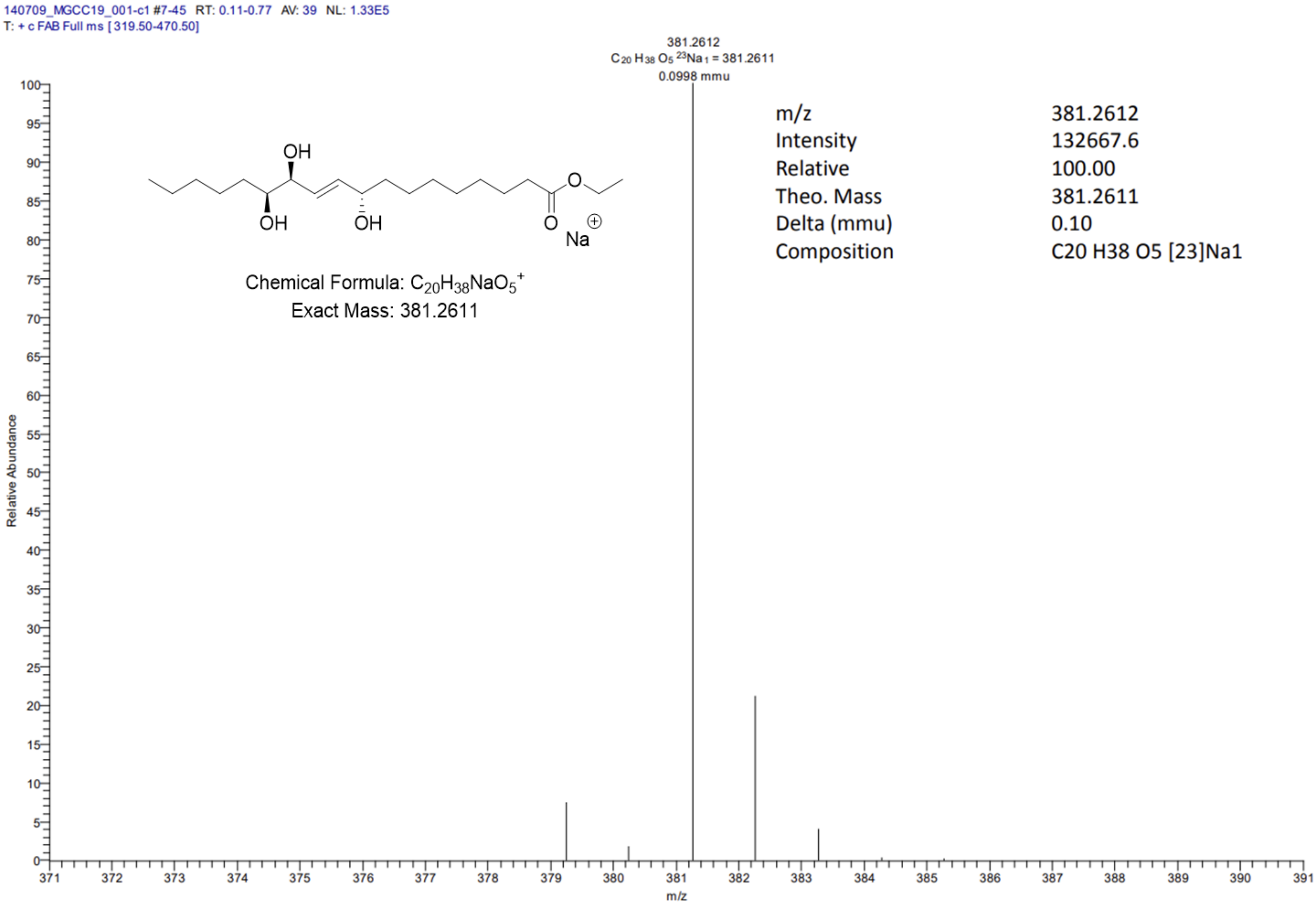

Figure S14. ^{1}H NMR spectrum of **3** in chloroform-*d* (700 MHz)

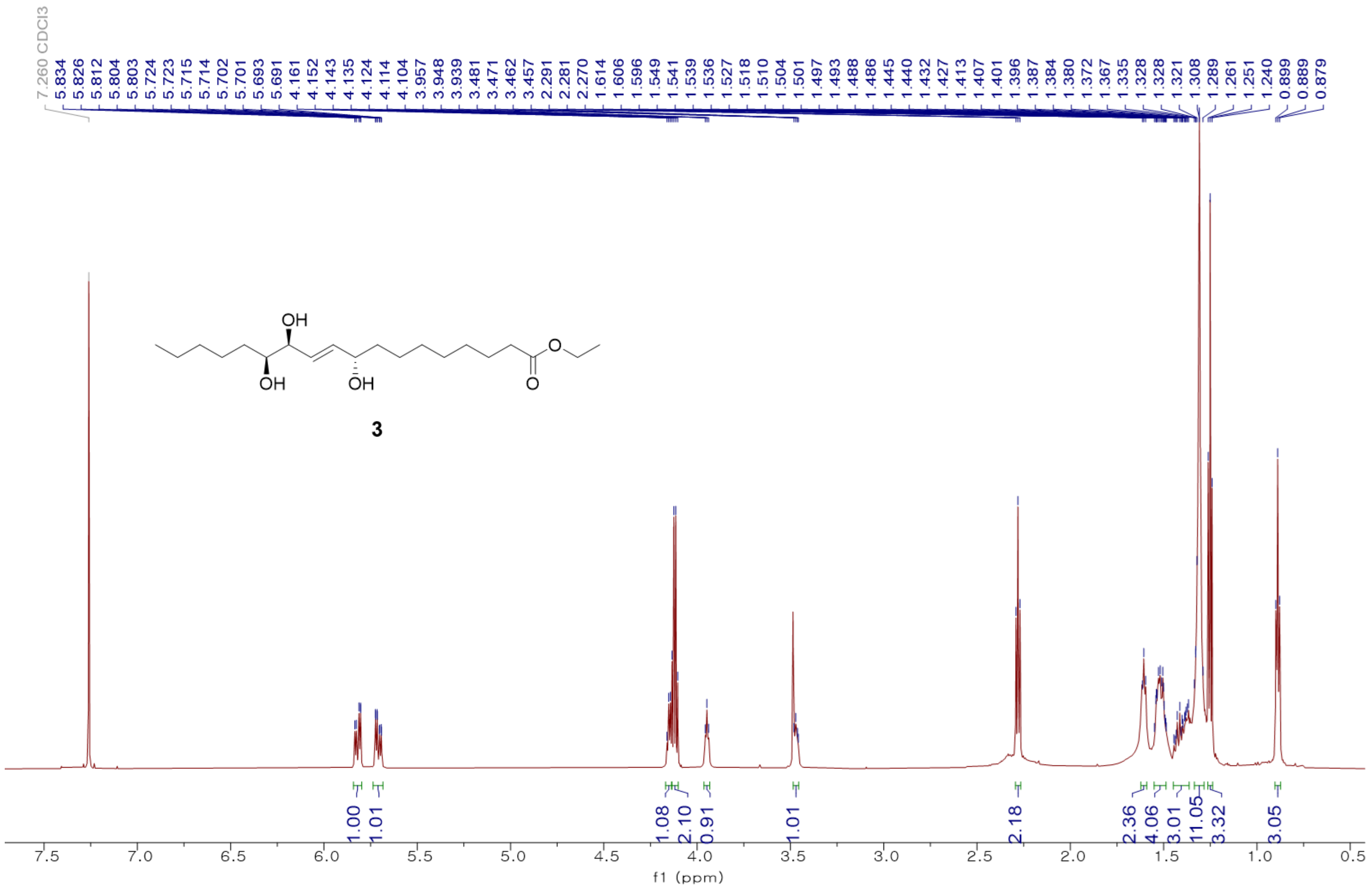

Figure S15. ^{13}C NMR spectrum of **3** in chloroform-*d* (175 MHz)

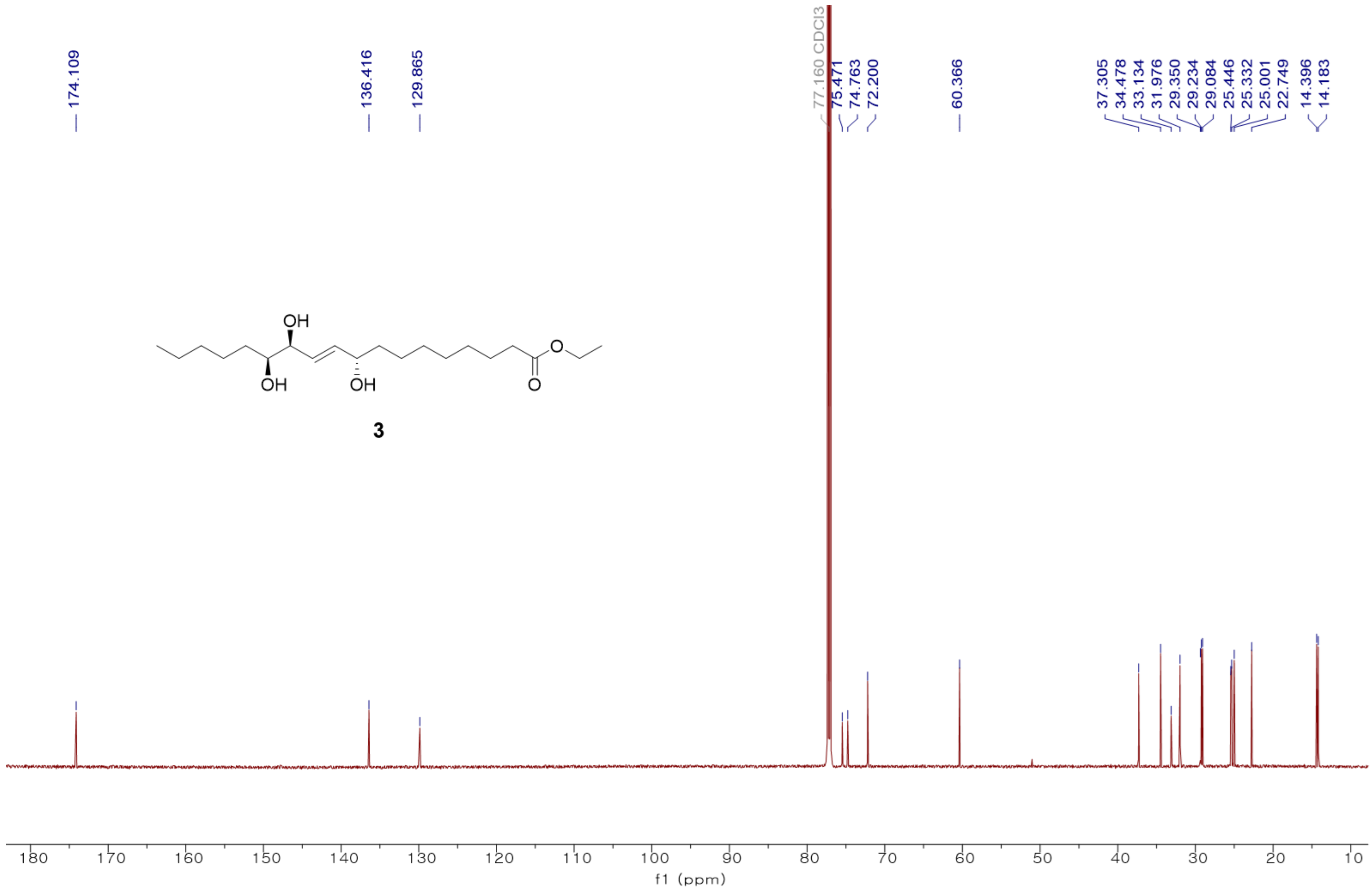

Figure S16. COSY spectrum of **3** in chloroform-*d*

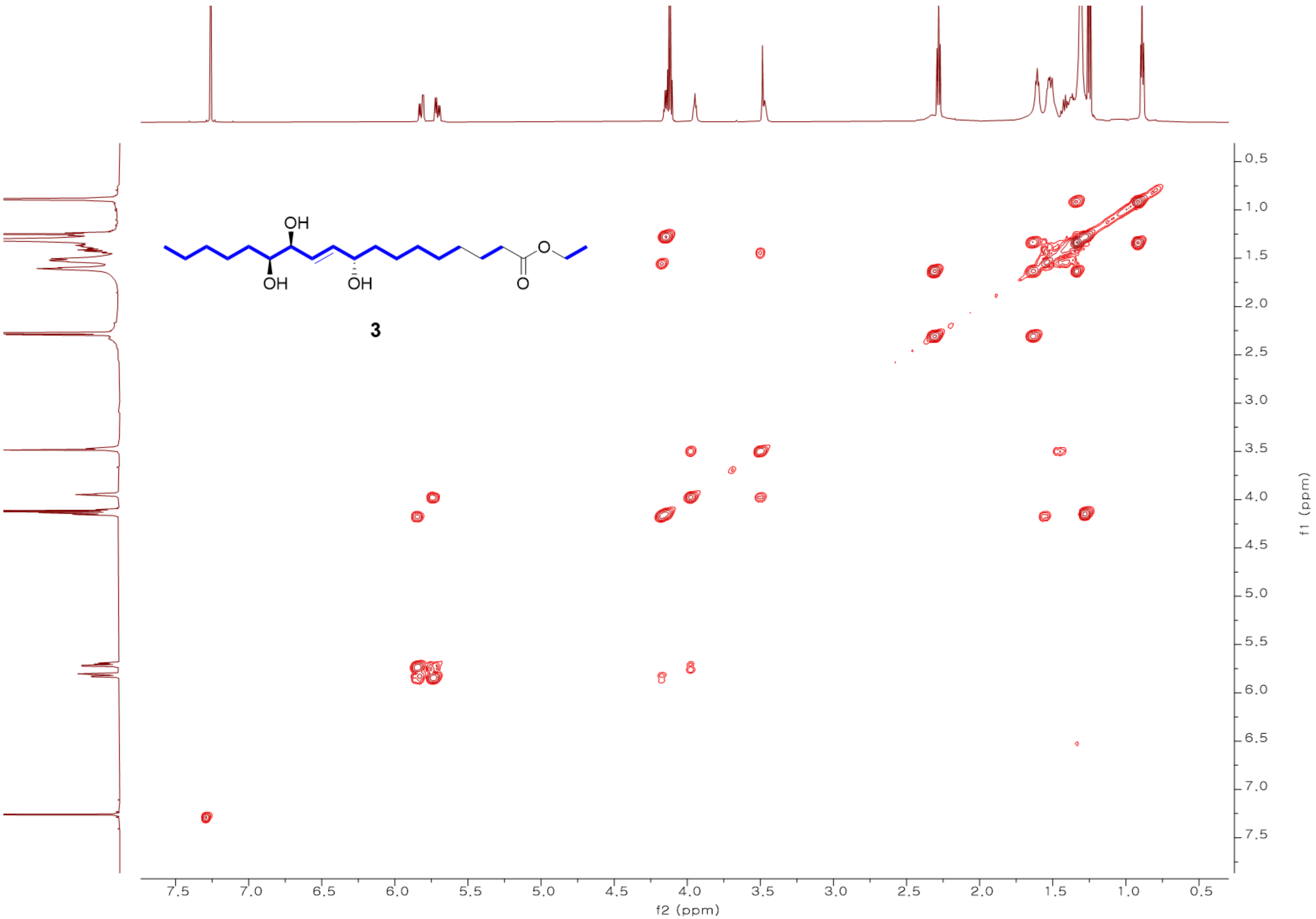

Figure S17. HSQC spectrum of **3** in chloroform-*d*

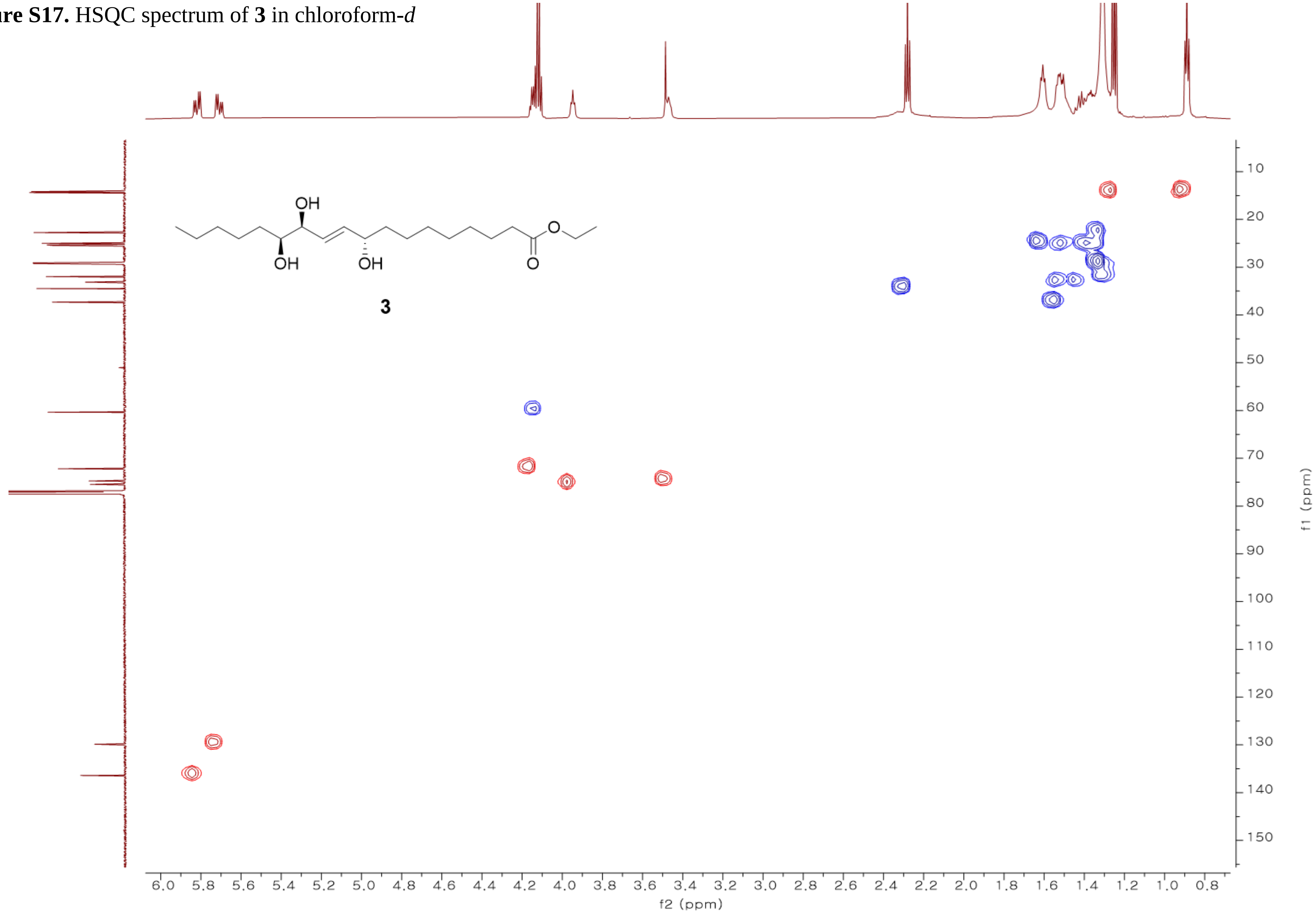

Figure S18. HMBC spectrum of **3** in chloroform-*d*

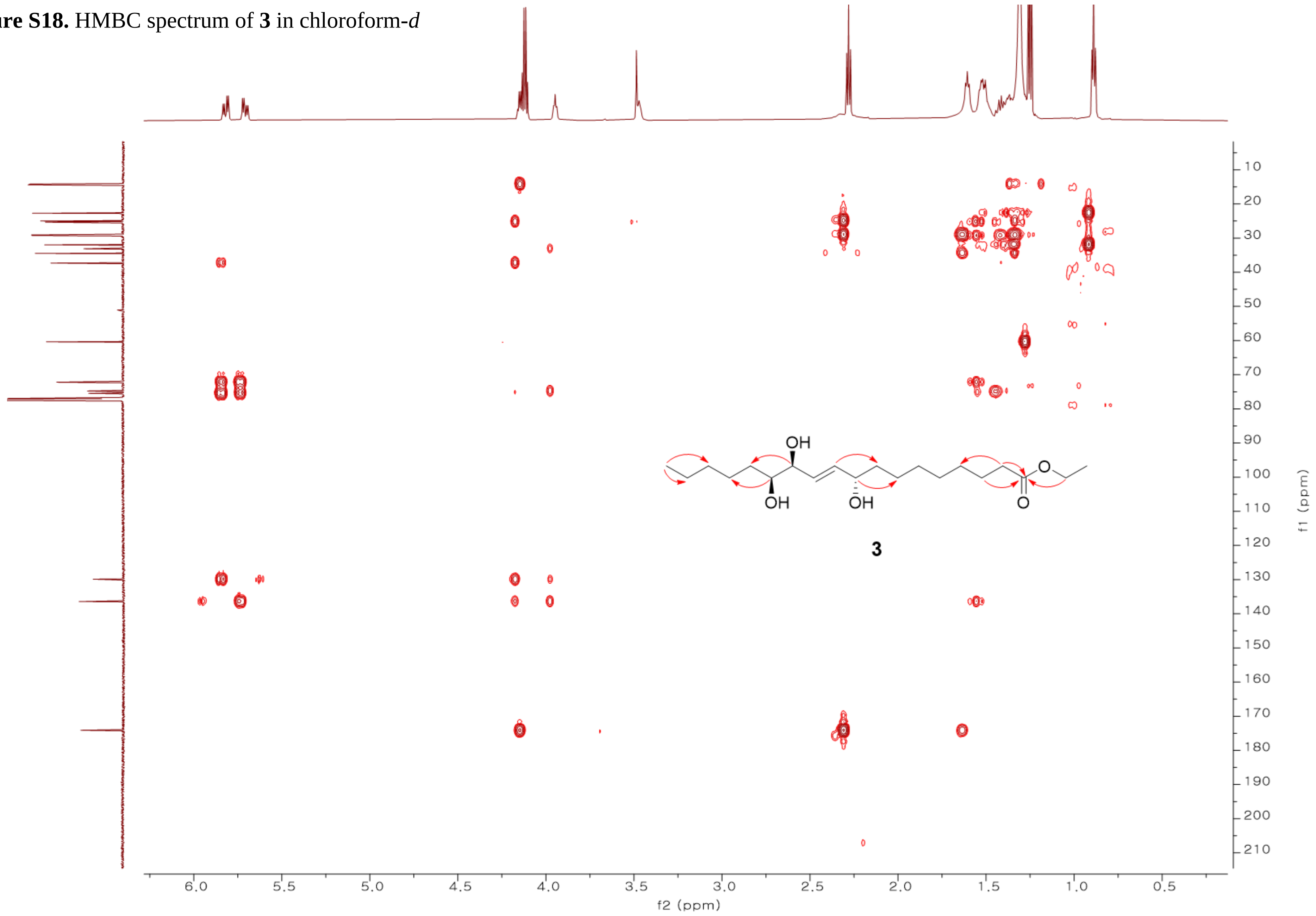

Figure S19. HRFABMS spectrum of **4**

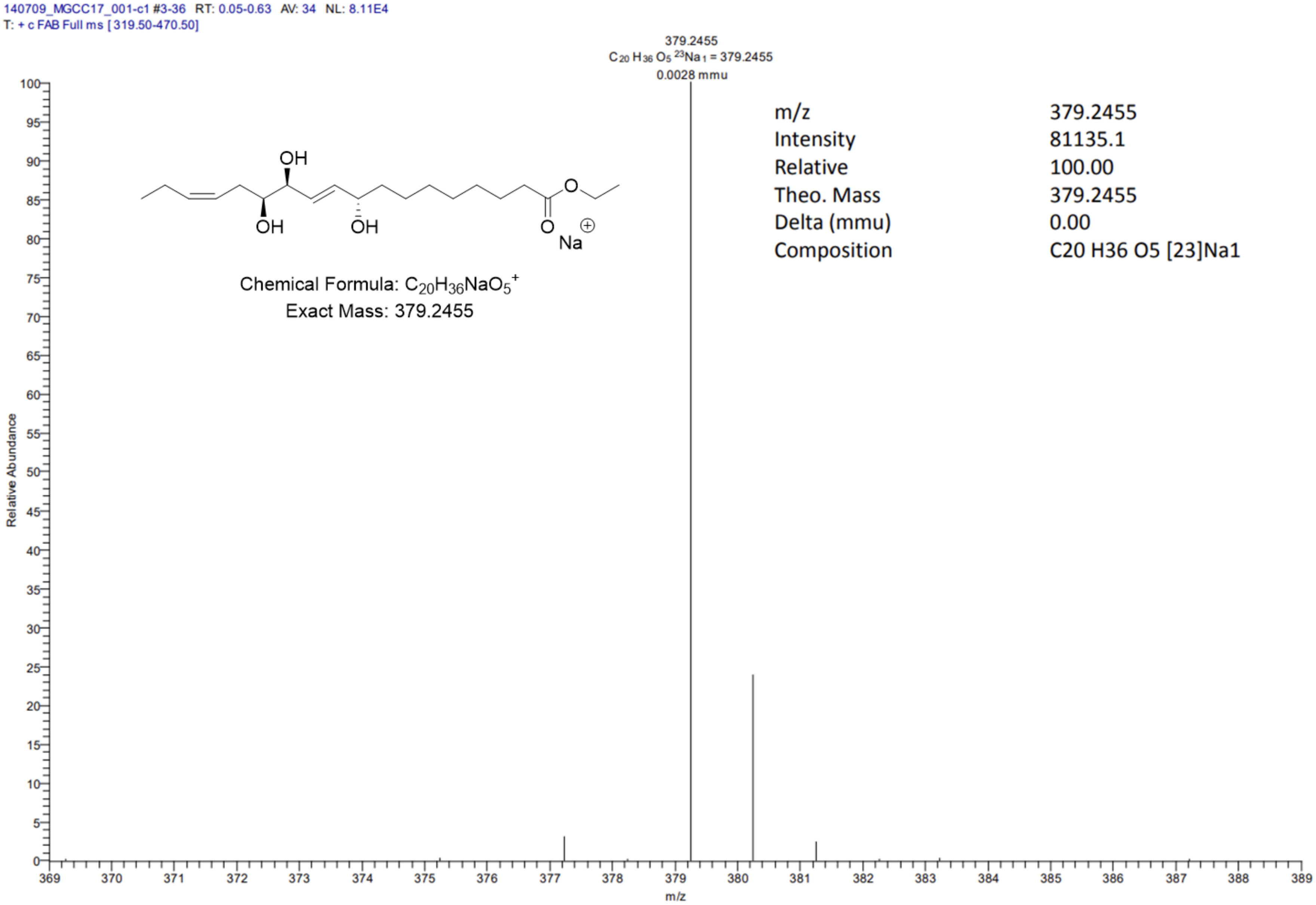

Figure S20. ^{1}H NMR spectrum of **4** in chloroform-*d* (700 MHz)

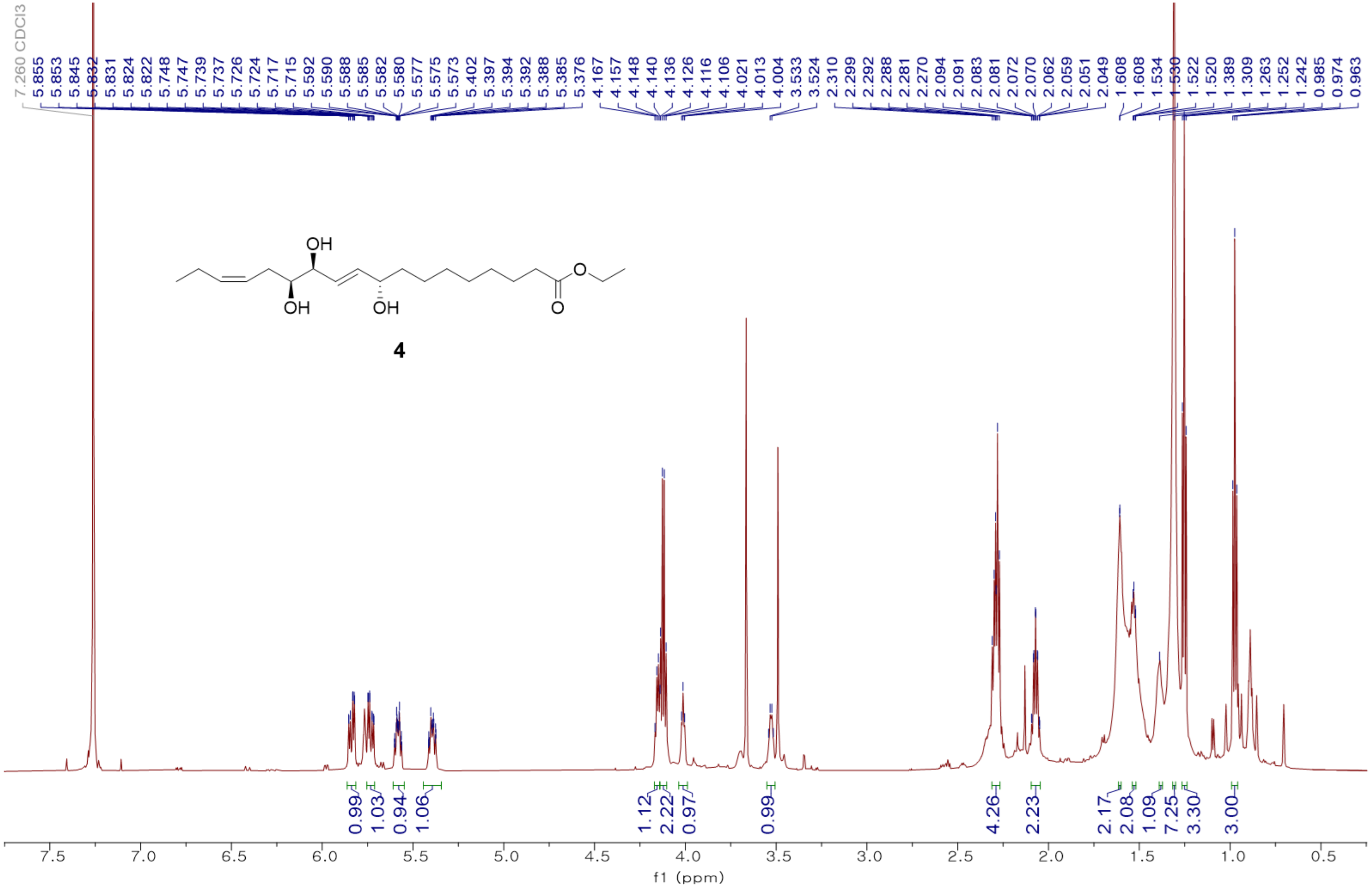

Figure S21. ^{13}C NMR spectrum of **4** in chloroform-*d* (175 MHz)

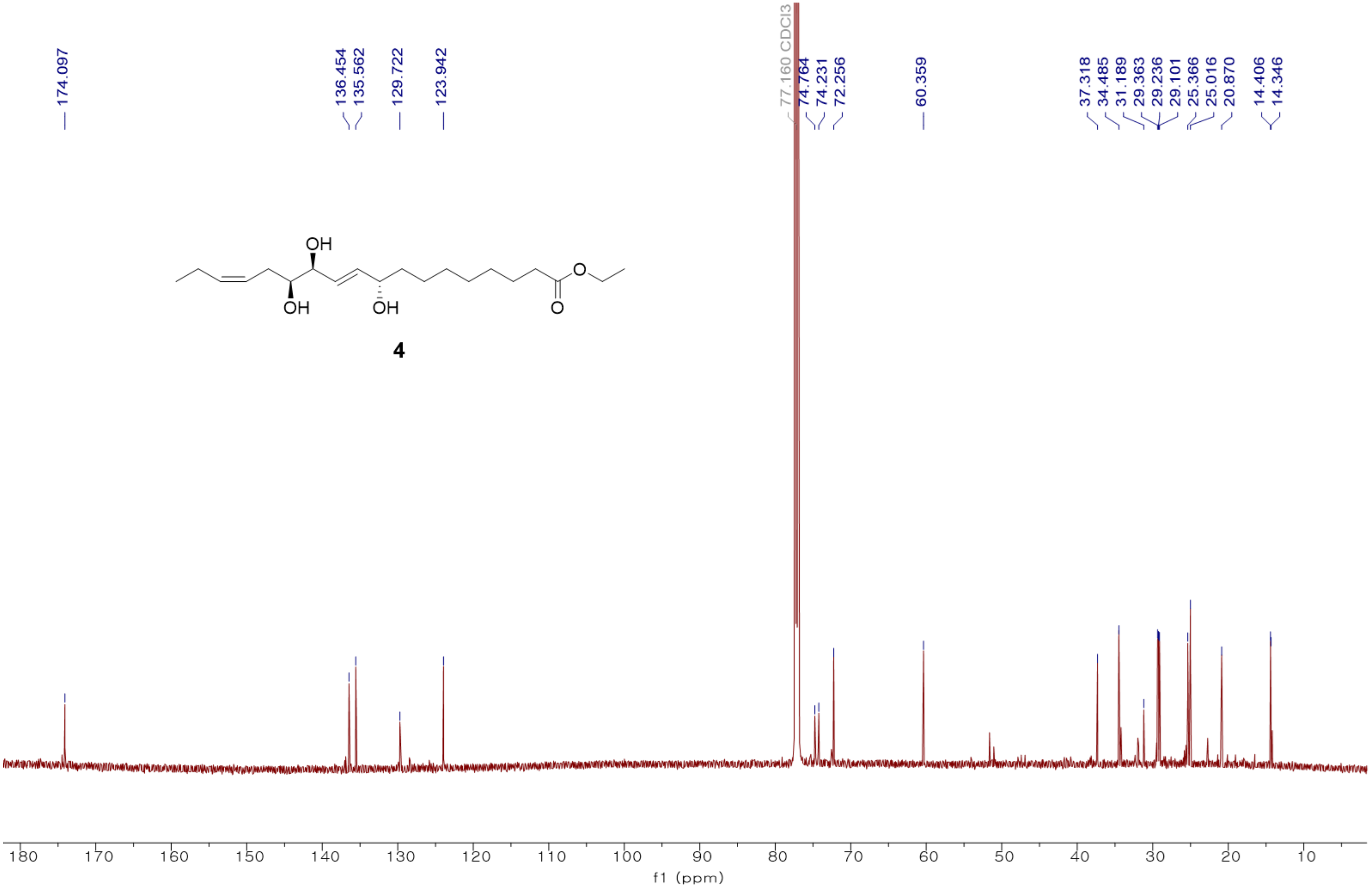

Figure S22. COSY spectrum of **4** in chloroform-*d*

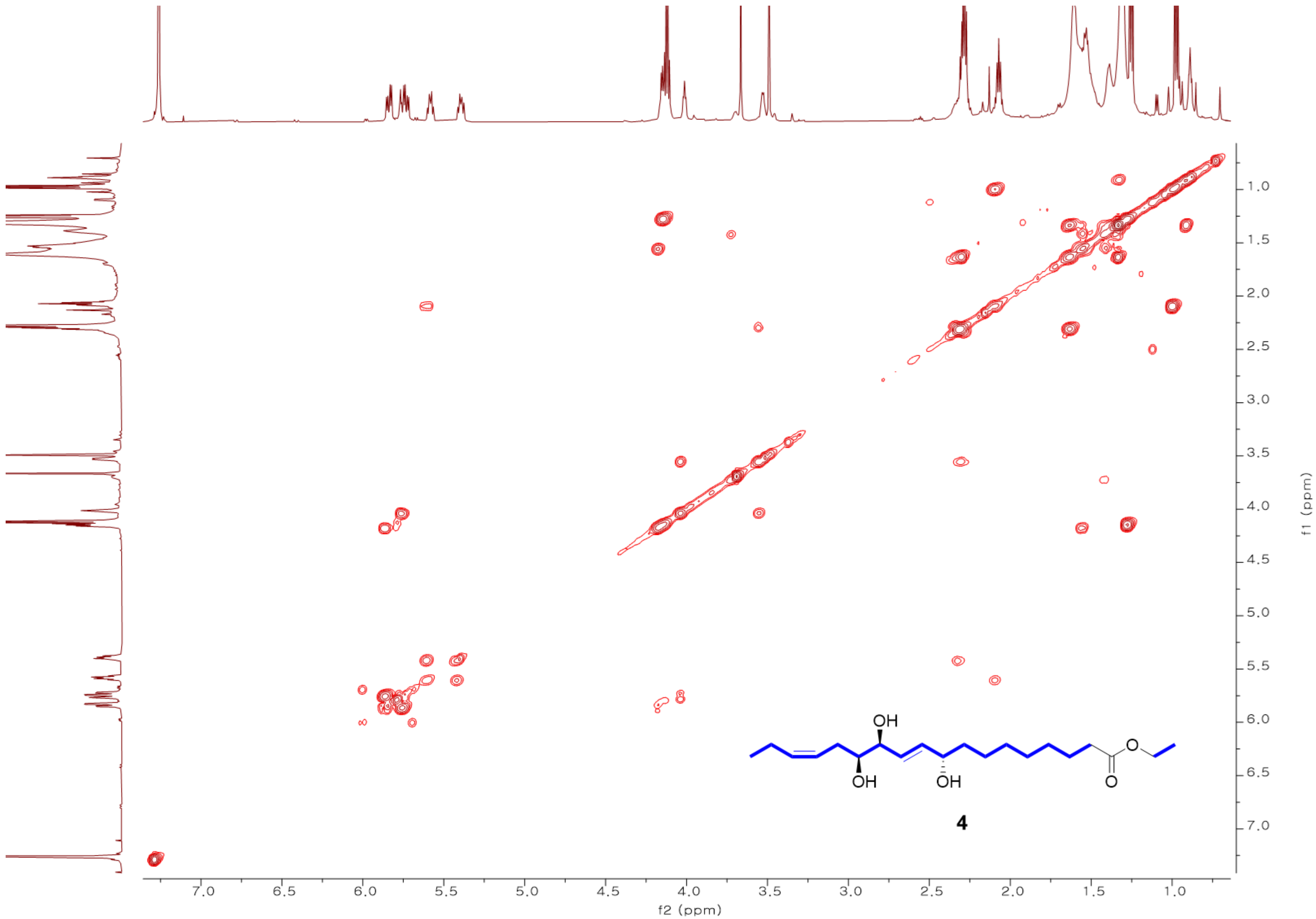

Figure S23. HSQC spectrum of **4** in chloroform-*d*

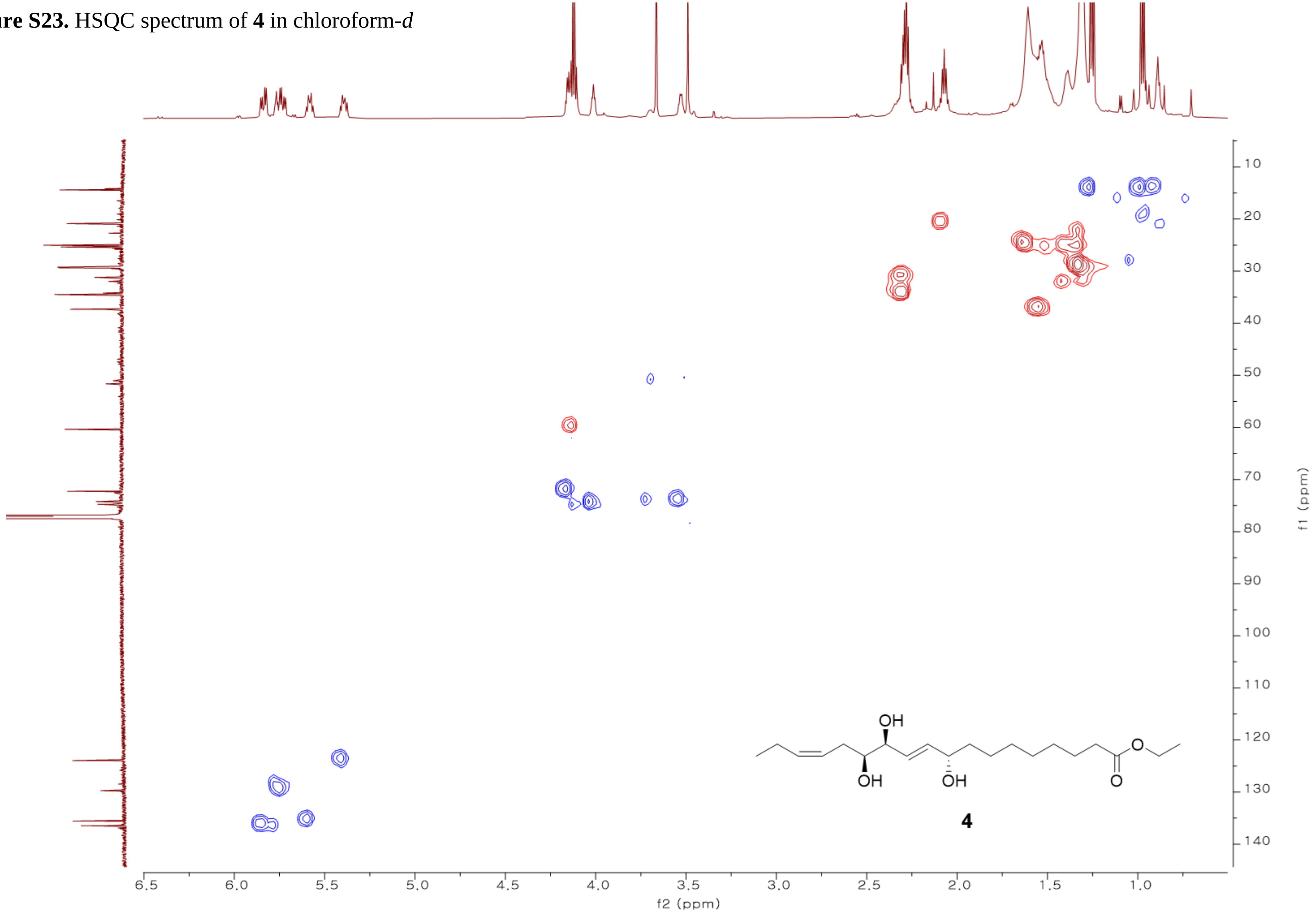

Figure S24. HMBC spectrum of **4** in chloroform-*d*

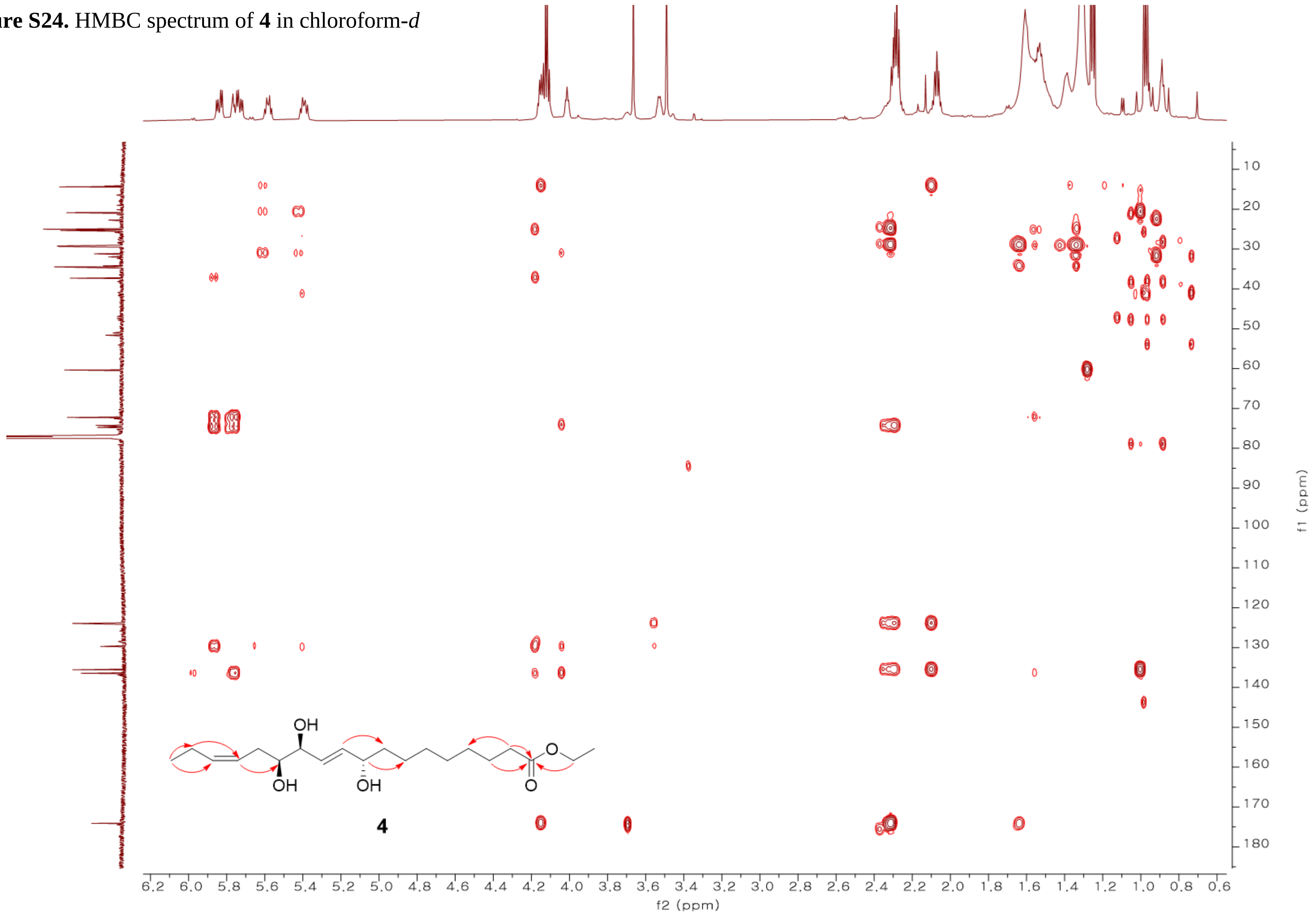

Supplement: Supplementary file 1 [file antioxidants-12-00284-s001.zip › antioxidants-2112674-supplementary.pdf]
